# The Triadic Principle of Biology: A Testable Hypothesis About Functional Closure in Living Systems

**DOI:** 10.3390/biology15211966

**Published:** 2026-11-01

**Authors:** Burim N. Ametaj

**Affiliations:** Department of Agricultural, Food and Nutritional Science, Faculty of Agricultural, Life and Environmental Sciences, University of Alberta, Edmonton, AB T6G 2P5, Canada; bametaj@ualberta.ca

**Keywords:** triadic principle, systems biology, biological organization, organizational closure, emergence, homeostasis, disease networks, biomarkers, therapeutic restoration, theoretical biology

## Abstract

Systems biology maps extensive networks but lacks a general principle identifying the minimum functional architecture of living organization. The Triadic Principle of Biology (TPB) proposes that stable, self-regulating biological systems require coordinated closure among three functionally distinguishable domains: Information, Energy/Material Transformation, and Interface/Boundary. These are categories of biological work rather than anatomical structures. This article states the claim, gives explicit rules for assigning a process to a domain and for fixing the system boundary, defines autonomy independently of the framework, and evaluates candidate manifestations in genetic coding, translation, cellular organization, epigenetics, development, physiology, evolution and disease against prospective admissibility criteria. The codon inequality provides one exact result, but it concerns code word length rather than functional domains and is not evidence for the TPB. The framework contributes a standardized functional vocabulary, criteria distinguishing genuine triads from arbitrary groupings, a taxonomy of failure modes, an operationalized closure index with explicit scoring functions and a worked example, and falsifiable predictions, including specified comparisons against rival decompositions such as sensing–regulation–action, supply–demand–control and Wicken’s exploitation–homeostasis dyad. Gánti’s chemoton is identified as the closest formal antecedent. Because these predictions remain untested, the article specifies the experiments and outcomes that would support, weaken or reject the principle.

## Simple Summary

Biology can identify countless components but still lacks a general rule explaining why similar functional arrangements recur across scales. This article proposes the Triadic Principle of Biology: stable, self-maintaining biological systems require three coordinated domains—Information, Energy/Material Transformation, and Interface/Boundary. These are functions, not necessarily three anatomical parts; many components may share a domain, and one component may contribute to several domains. A cell, for example, implements these functions through genetic and regulatory information, metabolism and bioenergetics, and its membrane and transport systems. The principle further proposes that disease may begin when one domain or coupling fails and then propagate through the connected system. It generates three main clinical predictions: the first failing domain should be identifiable; measurements spanning all three domains may outperform single-domain markers; and treatments that restore coordination may be more durable than single-target interventions. These claims remain unconfirmed. This article therefore defines the principle, distinguishes genuine triads from arbitrary groupings, proposes experimental tests, and specifies findings that would reject it.

## 1. Introduction: The Architectural Question in Biology

Three is not treated as a mystical number or a preference of classification, but, on the hypothesis examined here, as a candidate minimum at which biology separates information, transformation, and boundary-mediated exchange while keeping them causally coupled. A triplet codon converts a four-symbol nucleotide alphabet into a vocabulary large enough to encode the standard amino acids. Translation depends on a template, an adaptor, and a catalytic scaffold. Autonomous cellular life requires heritable organization, metabolic work, and a selective boundary. Vertebrate immune defense integrates barriers, innate execution, and adaptive memory. Complex disease can be described as unfolding through coupled regulatory, metabolic, and interface disturbances. The recurrence is striking, but recurrence alone does not establish a law, and several of these examples are tripartite only in a descriptive sense; none shows that three domains are biologically privileged. The scientific question is whether these examples express a common functional architecture that can be defined prospectively, tested against alternatives, and falsified.

Modern biology has been exceptionally successful at decomposition. It can identify molecular components, quantify thousands of transcripts and metabolites, map interaction networks, and resolve subcellular structures at remarkable spatial and temporal scales. Yet a catalog of parts does not by itself disclose the architecture that makes the parts a living system. Systems biology emerged in part to address this gap, emphasizing interactions, feedback, robustness, and emergence rather than isolated components [1]. Theoretical biology has pursued related questions through the closure of constraints [2,3], minimal-cell models [4], autopoiesis [5] and relational biology [6], and through the cybernetic and network-motif studies examined in Section 3.2. The Triadic Principle of Biology (TPB) is proposed within this intellectual lineage, but with a distinct claim: a broad class of stable, self-regulating biological systems is organized around a minimum core of three functionally differentiated domains—Information, Energy/Material Transformation, and Interface/Boundary. These three names are used without variation throughout; where the text refers to a domain, it refers to a category of biological work, not to a protein domain or to a domain of life in the taxonomic sense. A glossary of technical terms follows the abbreviations table at the end of this article.

The term “law” requires care. Biological regularities are historically contingent, multiply realized, and often conditional. They need not resemble universal equations of fundamental physics to support explanation, prediction, counterfactual reasoning, and intervention. Philosophers of biology have accordingly distinguished degrees and dimensions of lawfulness rather than treating lawhood as all-or-none [7]. The TPB uses “law” in this pragmatic biological sense: a robust, scale-transcending organizational generalization that generates risky predictions and survives specified counterexamples. The manuscript therefore does not declare a theorem by rhetorical force. It states a candidate law, defines its domain, separates mathematical results from biological inference, and identifies observations that would require revision or rejection.

The TPB is specifically a principle of biology. Similar triadic arrangements may be useful when analyzing non-biological systems, but the Sun–Earth–Moon relation, physical dimensions, philosophical triads, or other examples outside life are not used here as evidence for a biological law. The claim of broad applicability refers to living organization and its pathological failure. Disease is consequently not an afterthought. It provides one of the strongest opportunities to test the TPB because it reveals what happens when a domain becomes deficient, hyperactive, bypassed, hijacked, uncoupled, or collapsed. The working proposition is stated as a hypothesis and not as a finding: disease can become triadic disruption, and durable therapy may require restoration of triadic function. This does not mean that every disease starts simultaneously in three places or that every treatment must contain three drugs.

One clarification belongs at the outset, because the framework is a claim about relationships rather than about counting. Organizational closure is the condition in which the parts of a system make each other possible: each helps maintain the circumstances the others require, so that none is simply upstream of the rest. The article then proceeds in five steps. First, it defines the three domains and states the rules that govern their use: how a process is assigned to a domain, how the system boundary is fixed, and what autonomy means independently of the framework. Second, it places the TPB relative to Gánti’s chemoton, Wicken’s thermodynamic program and the criticism this program attracted, autopoiesis, organizational closure, and network theory, with the fuller historical record given in Supplementary Section S1. Third, it examines the mathematical rationale, separating one exact conditional result from formal arguments that motivate but do not establish the hypothesis, and it operationalizes the closure index. Fourth, it evaluates representative biological manifestations across molecular, cellular, developmental, physiological, evolutionary, and pathological levels. Finally, it presents falsification criteria and a prospective research program. The central question is not “Where can three be found?”; it is whether a predefined triadic functional model explains and predicts biological organization better than dyadic, tetradic, alternative-triadic, or unconstrained alternatives—a question posed here and answered only in the sense of specifying how it could be settled.

### Scope, Claim, and Evidentiary Status

Because the framework is broad and the qualifications governing it are easily lost inside the sections they qualify, they are stated once here and are not restated in full thereafter.

What is claimed. The TPB is a hypothesis about functional architecture, not an observation about the recurrence of a number. The claim is that a broad class of stable, self-regulating biological systems requires coordinated closure among three functionally differentiated domains, and that progressive loss of this closure provides a general model of pathogenesis. The claim is not that biological objects come in threes, that every system contains exactly three physical parts, or that finding three of anything supports the principle. Where an example is numerically attractive but architecturally weak, this article says so and records the failure rather than absorbing it. Three kinds of example are kept apart throughout: examples that are tripartite only descriptively (three-level endocrine axes, germ layers, sensor–integrator–effector loops, the tripartite synapse), candidate TPB mappings that remain provisional (translation, the cell, epigenetic regulation, immune defense, disease), and demonstrated TPB triads, of which this article contains none. A descriptive tripartition is never counted as evidence of irreducible TPB structure.

Functions, not structures. The three domains are categories of biological work. Each may be realized by many components, one component may contribute to more than one domain, and no anatomical mapping is asserted as necessary anywhere in this article, and where organelles or organs are named they are illustrative instances of one implementation and are labeled as such. Because this latitude is what makes post hoc assignment possible, Section 2.3 states an explicit rule for assigning a process to a domain and Section 2.4 an explicit rule for fixing the system boundary; these rules, rather than the definitions themselves, are what the framework relies on to remain falsifiable.

What the mathematics does and does not do. Section 3 asks whether any reason independent of biology favors three. One exact result is available, concerning the minimum length of a genetic code word, and because it concerns word length rather than functional domains it is not evidence for the TPB. The remaining formal arguments establish only that a third element makes possible operations two elements cannot perform. No mathematical necessity of three functional domains is claimed anywhere in this article, and none is proved.

Evidentiary status. None of the predictions in Section 9 has been tested here, and no value of the closure index reported in this article is derived from measurement. What is offered is a candidate architecture, prospective criteria for applying it without circularity, a definition of autonomy that does not presuppose it, a vocabulary for its failure, an operationalized index, and a specification of the observations that would require it to be abandoned. Throughout, this article distinguishes three registers explicitly: what the cited literature establishes, what the present author has previously proposed, and what the TPB predicts and has not yet been tested.

Citation balance and self-citation. Several of the applications in Section 7 and Section 8 rest partly on work the present author has published: the Keto–Inflammatory Network (Section 7.4), the Lameness Triad and the Susceptibility–Resilience Gate (Section 7.5), Stratodynamics (Section 8), and two empirical studies of transition cow immunity and metabolism on which the dairy example in Section 7.3 draws. These are cited because they are the source of the specific proposals being extended, and not because they establish them. Where a claim depends on one of them the text says that the present author has proposed it; where the underlying physiology is independently documented, independent sources are cited alongside it. No claim in this article is treated as established on the strength of the author’s own publications, and a reader who set all five aside would find the architectural argument in Section 2, Section 3 and Section 9 unaffected, since none of it depends on them.

## 2. Conceptual Foundations and Definitions

### 2.1. Functional Domains Are Not the Same as Physical Components

The TPB concerns functional domains, not a literal count of anatomical objects. A eukaryotic cell contains a nucleus, mitochondria, endoplasmic reticulum, Golgi apparatus, lysosomes, peroxisomes, cytoskeleton, ribosomes, and many additional structures; a prokaryotic cell lacks a nucleus and mitochondria. Both can nevertheless implement the same broad requirements: preservation and deployment of biological information, energetic and material transformations, and selective exchange with the environment. The TPB node is therefore a functional domain that may be distributed across many components, and one component may contribute to more than one domain. Mitochondria, for example, transform energy, generate signaling metabolites, participate in apoptosis, and influence nuclear gene regulation [8]. Asked instead what a car must do, one would say that it must convert stored energy into motion, control this motion, and protect its occupants—and a single component such as the engine block contributes to more than one of these, while the first requires dozens of parts acting together. The TPB counts in the second way: when it says a cell has three domains, it claims that three kinds of work must be underway, however many structures perform them. Because this latitude is precisely what makes post hoc assignment possible, Section 2.3 states the rule by which a process is assigned to a domain, and this rule—not the definitions below—is what the framework relies on to avoid circularity.

**Figure 1:**
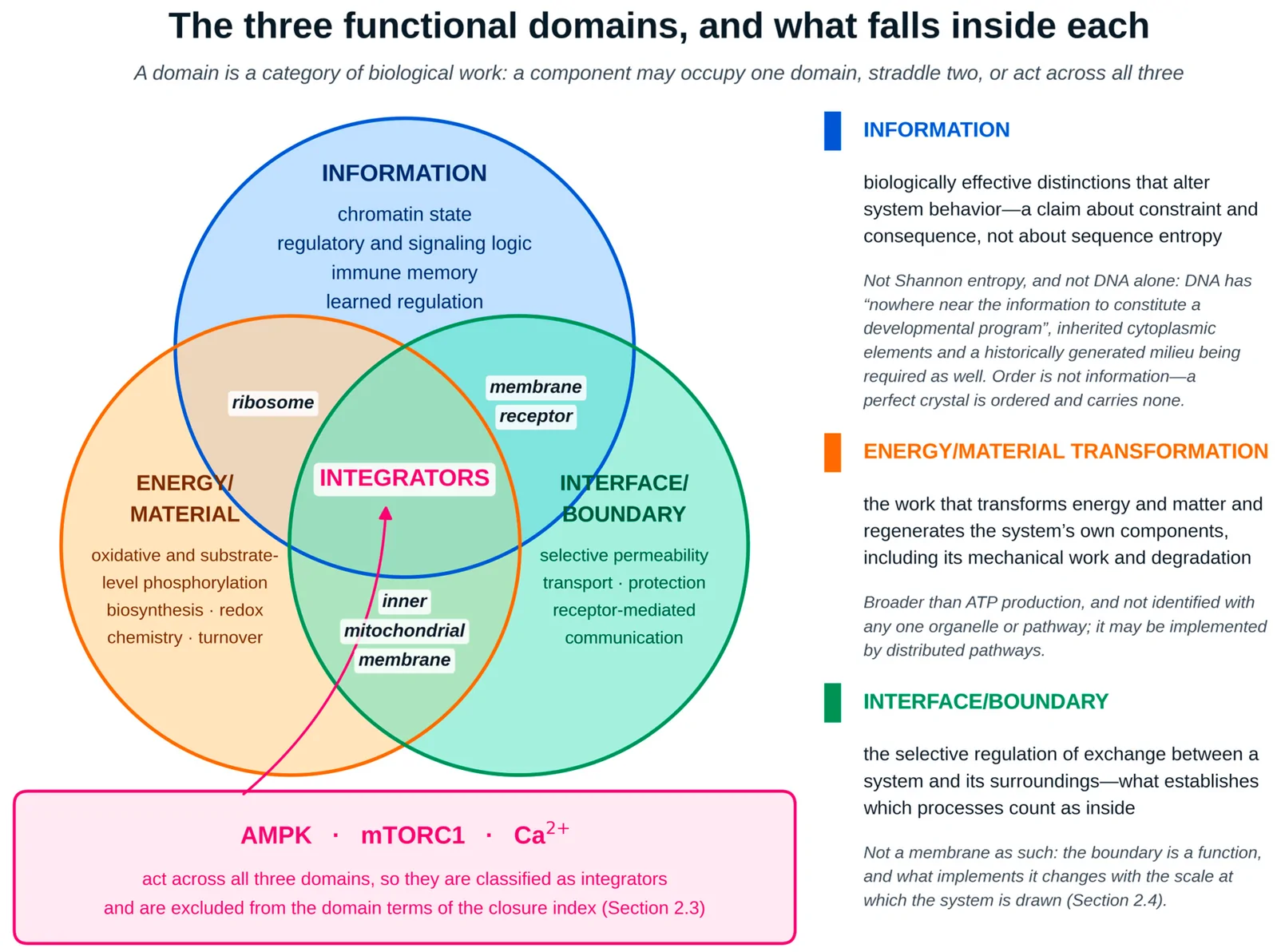
The three functional domains, and what falls inside each. The domains are categories of biological work rather than anatomical parts, so they are drawn as overlapping regions rather than as separate boxes. Processes in the non-overlapping regions belong predominantly to one domain. Processes lying in a pairwise overlap discharge both functions and cannot be assigned to one without loss: the ribosome reads a sequence and performs biosynthetic work; a membrane receptor senses the exterior and sets the transcriptional state; the inner mitochondrial membrane maintains a proton gradient, which is energy transduction carried out across a boundary. Processes in the central region act across all three domains and are therefore classified as integrators under the rule in Section 2.3, entering the closure index through the coupling terms rather than through any domain term. The right-hand column gives each definition and, beneath it, the exclusion that disciplines it. The figure summarizes the definitions given in this section and does not introduce any claim not made there; the placements shown are illustrative and remain subject to the property-first rule in Section 2.3 and Section 2.4. The distinction between biological information, order and thermodynamic entropy, including the quoted limitation of DNA as a complete developmental program, follows Wicken [9].

The Information domain includes the storage, interpretation, transmission, and adaptive modification of constraints that give a system identity and direct its operations. At different scales it may be implemented by nucleic acid sequence, chromatin state, signaling logic, immune memory, or learned regulatory organization. Information in the TPB is not restricted to Shannon entropy or to DNA sequence, and this restriction is deliberate rather than loose. Wicken argued that the Shannon formula does not generalize thermodynamic entropy at all, because the two rest on different probabilistic foundations: a thermodynamic macrostate is an ensemble of microstates among which a system genuinely fluctuates, whereas a specified sequence is a completed structure with no such ensemble and therefore zero configurational entropy. What the Shannon formula measures, according to Wicken, is the complexity of structural relationships, and he distinguished this in turn from order, which is an internally imposed limitation on alternatives and which a perfectly ordered crystal exhibits without carrying information at all [9]. The TPB adopts this discipline. Information here denotes biologically effective distinctions that alter system behavior, which is a claim about constraint and consequence rather than about sequence entropy. The same argument supports treating the domain as distributed rather than genomic, since Wicken concluded that DNA alone possesses “nowhere near the information to constitute a developmental program” [9], inherited cytoplasmic elements and a historically generated milieu being required as well. The Energy/Material Transformation domain includes metabolism, redox chemistry, biosynthesis, mechanical work, degradation, and the energetic execution of biological programs. It is broader than ATP production and may be implemented by distributed pathways. The Interface/Boundary domain includes selective permeability, sensing, transport, protection, receptor-mediated communication, and the regulation of exchange between a system and its surroundings. Figure 1 summarizes what each domain covers, what each excludes, and how a single component may occupy one domain, straddle two, or act across all three.

**Figure 2:**
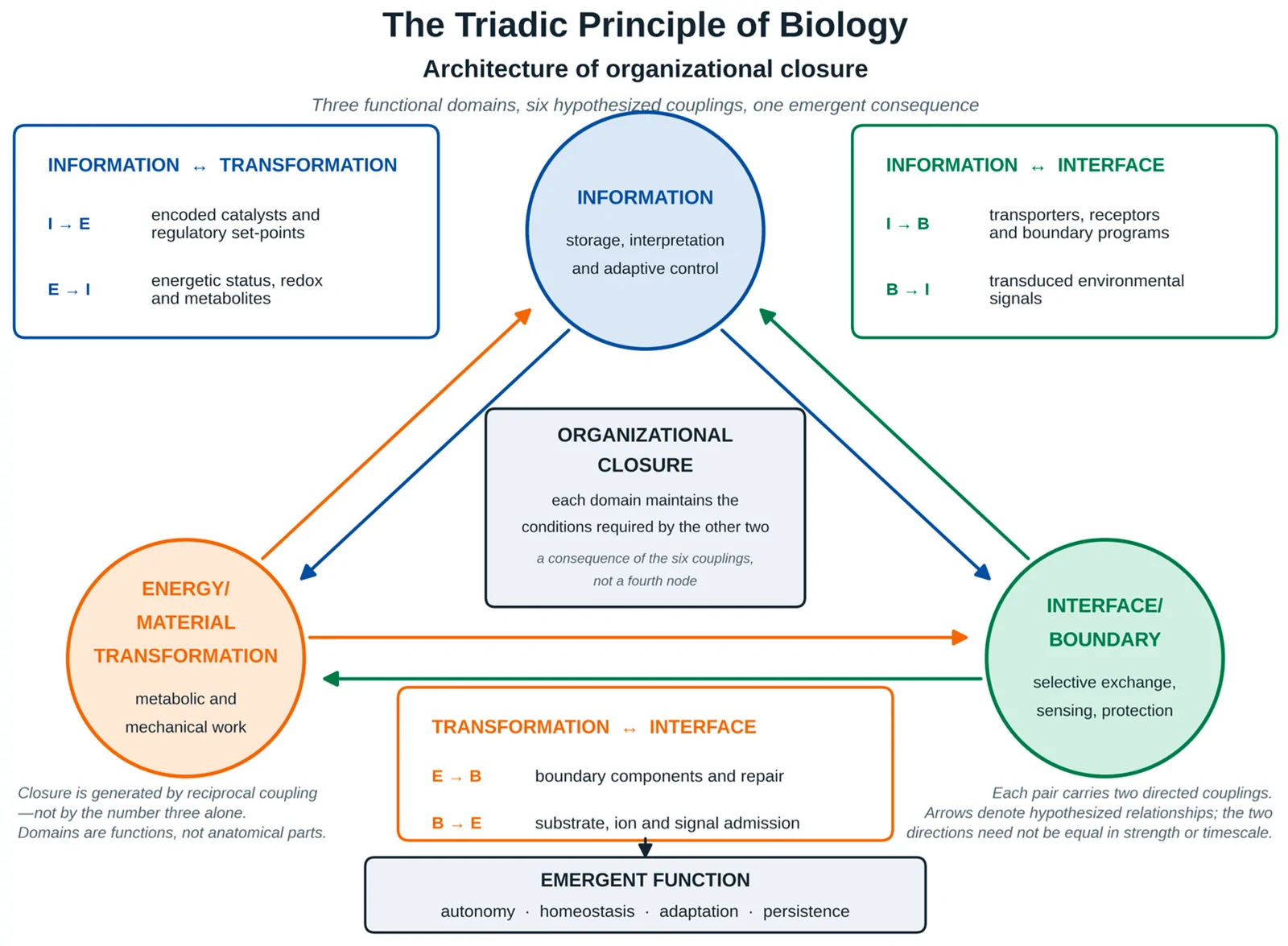
Architecture of organizational closure in the Triadic Principle of Biology. Information, Energy/Material Transformation and Interface/Boundary are proposed to be functionally distinct and reciprocally coupled, with each pair linked in both directions, giving six couplings in total. The six arrows denote hypothesized causal relationships and are not asserted to be of equal strength, equal timescale, or symmetrical: the influence of i on j need not equal the influence of j on i, and Section 3.3 estimates the two directions separately where the sampling design permits. Organizational closure is a consequence of these couplings rather than a fourth component. Closure—not the number three—is what is proposed to generate the emergent properties of autonomy, homeostasis, adaptation and persistence. The figure states a hypothesis to be tested by the program in Section 9, not an established architecture. Arrowheads indicate the proposed direction of influence. Blue arrows originate in Information, orange arrows in Energy/Material Transformation, and green arrows in Interface/Boundary. The black downward arrow denotes the proposed emergence of system-level function from the coupled architecture, rather than an additional coupling or domain.

**Figure 3:**
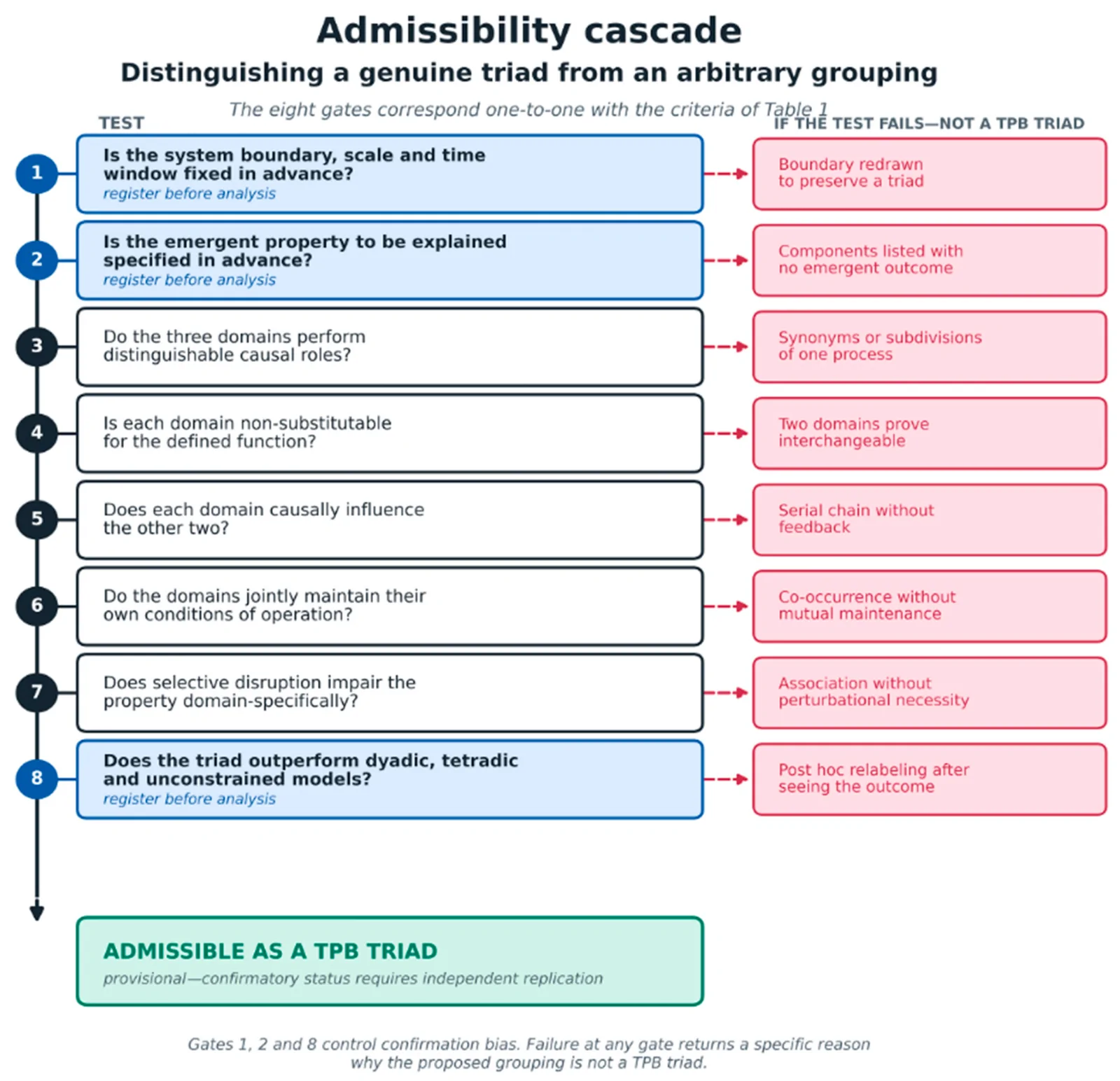
Admissibility cascade: distinguishing a genuine triad from an arbitrary grouping. The cascade is the operational form of Table 1. Gates 1, 2 and 8 are the confirmation bias control and must be registered before the data are examined: a triad identified after the outcome is known is exploratory, not confirmatory. Registration under Gates 1 and 2 fixes the system boundary and time window (Section 2.4), the emergent property, the domain assignment including any weighted memberships (Section 2.3), the indicator set and viable ranges, the steepness parameter κ, the coupling reference pattern and tolerance parameters α and β, and the number m and identity of the essential couplings (Section 3.3). Gates 3–7 test the substantive architectural claims of functional differentiation, non-substitutability, reciprocal coupling, organizational closure and perturbational necessity, and each requires a pre-declared decision rule: for Gate 4, the effect size and time window within which a domain must fail to be judged non-substitutable; for Gate 5, the minimum coupling magnitude and maximum permissible lag; for Gate 6, the evidence required that the domains jointly maintain their own conditions of operation; and for Gate 7, the magnitude of domain-specific impairment that counts as perturbational necessity. Gate 8 requires that the triadic model outperform dyadic, tetradic, alternative-triadic and unconstrained alternatives under matched parameterization rather than merely fit the data. Failure at any gate returns a specific diagnosis of why the proposed decomposition is not a TPB triad, which is what distinguishes the framework from post hoc pattern matching.

These domains are logically distinguishable but materially interpenetrating. Biological information cannot be maintained without energetic work; metabolism depends on encoded catalysts and regulatory states; a boundary is produced and repaired by metabolism; and the boundary controls which substrates, signals, and perturbations reach the other domains. The essence of the TPB is therefore not three isolated compartments but reciprocal dependence. Figure 2 summarizes this architecture, and Figure 3 sets out the admissibility cascade used to apply it.

Table 1 summarizes the prospective criteria used in this assessment.

### 2.2. Why These Three Domains Rather than Other Tripartitions?

A framework proposing a privileged decomposition must say what would make a rival decomposition wrong, and the most serious challenge to the TPB is not that biology resists division into three but that it admits several equally coherent divisions into three. Two rivals are the strongest. Sensing–regulation–action, familiar from control theory and behavioral physiology, partitions biological activity into perception of state, computation of a corrective response, and its execution [10]. Supply–demand–control, familiar from metabolic control analysis, partitions flux into the processes that produce a metabolite, those that consume it, and those that set the balance [11]. Neither is a straw man, and either could be applied to most of the systems surveyed in Section 4, Section 5 and Section 6.

The answer proposed here is not that these decompositions are incorrect but that they decompose a process rather than the conditions of persistence. Sensing, regulation and action are three stages of a single control operation; a system executing all three faultlessly still dissolves if nothing maintains the boundary across which sensing occurs or regenerates the catalysts on which regulation depends. Supply–demand analysis, as its authors set it out, partitions metabolism into a supply block and a demand block linked by a common intermediate or a moiety-conserved cycle, and quantifies how flux control and concentration control are distributed between them. Its central result is that the two are mutually exclusive: a block that controls flux thereby loses influence over the homeostatic maintenance of the linking metabolite, which becomes the other block’s function [11,12]. This is an analysis of control distribution within an already-existing metabolic system [11,12], and, based on the reading offered here and not one these authors state, it presupposes the catalysts, the compartment and the evolved set-points on which such control operates. The same authors require that regulatory performance always be measured in terms of a specified function [11], which is the rule the TPB adopts in Section 2.4 when it fixes the emergent property before the system boundary. The TPB domains are proposed instead as the three aspects that must be underway for a system to remain the kind of thing that can sense, regulate, act, supply, demand or control: a set of constraints that persists and can be deployed, work that regenerates the system’s own components, and a selective boundary that establishes what counts as inside. Based on this reading the rival tripartitions describe operations that a triadically closed system performs rather than the conditions of its persistence. This reading is itself a claim, and it makes a prediction: if the two partitions describe different things, they should not predict the same perturbation outcomes equally well.

A third rival deserves separate treatment because it is not a tripartition at all and because it comes from the same author whose distinction between information and entropy is adopted in Section 2.1. Working from thermodynamics, Wicken derived two general meta-functions for biology rather than three: exploitation, in which the dissipation of free energy is coupled to the generation, stabilization and propagation of the organizational relationships that define the self; and homeostasis, the preservation of the self as an internally referential seat of activity through feedback that holds its essential relationships within allowed tolerances [13]. This is a serious dyadic alternative, published, motivated independently of any interest in the number two, and it maps onto the TPB without remainder in one direction: exploitation is Energy/Material Transformation, and homeostasis subsumes what the TPB separates into Information and Interface/Boundary. The question the TPB raises against it is whether the subsumption is legitimate. The claim made here is that homeostasis conflates two functions that can fail independently and with distinguishable signatures—a lesion in regulatory set-points with barriers intact, and a barrier breach with set-points intact—and that a scheme with a single homeostatic function cannot distinguish them because both present as homeostatic failure. This is the same individuation criterion applied in the subsection “Is Energy/Material Transformation Too Broad to Be One Domain?” within Section 2.2, applied here in the direction that could cost the TPB a domain rather than add one. If the two failure modes prove not to be distinguishable under matched perturbation, the TPB should collapse to Wicken’s dyad, and Section 9.2 names exploitation–homeostasis among the required comparators for exactly this reason.

This is an argument, and arguments of this kind are exactly what the framework elsewhere refuses to accept as evidence. It is therefore restated as an experiment. The discriminating test is not which vocabulary sounds more fundamental but which decomposition, fixed in advance and matched for parameter count, better predicts the consequences of selective perturbation: recovery time, variance around the viable state, hysteresis, and persistence under a second challenge. If a sensing–regulation–action partition of the same system predicts these as well as or better than the TPB partition, the TPB’s claim to describe a distinct and deeper level is empirically idle, and the framework should be withdrawn in favor of the simpler description. Section 9.2 specifies this comparison alongside the dyadic and tetradic comparisons, and Section 9.3 requires that biomarker panels drawn from the rival tripartitions be included among the size-matched alternatives. Until these comparisons are performed, the priority of these three domains over other tripartitions is asserted and not shown.

#### Is Energy/Material Transformation Too Broad to Be One Domain?

A second and more specific objection concerns the comparability of the domains. Energy/Material Transformation encompasses metabolism, redox chemistry, biosynthesis, degradation and mechanical work, and is on any reasonable accounting a larger category than Information or Interface/Boundary. Why is it one domain rather than two—say, energy transduction and material synthesis—and if the domains are not comparable in scope, what does their number signify?

The criterion is non-substitutability applied within a candidate domain rather than only between domains. Two proposed domains are genuinely distinct only if each can fail while the other remains intact and the two failures produce distinguishable signatures. Energy transduction and biosynthesis do not currently satisfy this. Biosynthesis is thermodynamically and kinetically downstream of energy transduction in every cellular system characterized to date [14,15]: abolishing oxidative and substrate-level phosphorylation abolishes biosynthesis, whereas blocking an individual biosynthetic pathway leaves energy transduction intact. The relationship is one of asymmetric dependence on the timescale of an acute perturbation rather than of reciprocal coupling, and criterion 5 in Table 1 excludes asymmetric dependence as a domain boundary. The qualification matters: over the timescale of turnover the dependence is reciprocal, since energy transduction itself requires biosynthetically produced complexes, cofactors and lipids, so the criterion is applied on the timescale of the specified emergent property. What distinguishes the biosynthesis case is the absence, to date, of a demonstrated lesion that abolishes material synthesis while leaving energy transduction intact and produces a distinguishable failure signature. Information and Interface, by contrast, can each be selectively degraded with the other intact—a transcriptional lesion with membranes intact, a barrier breach with regulatory programs intact—and produce different failure signatures, which is what qualifies them as separate domains under the same rule. One complication should be stated here rather than left for a critic to raise. Metabolic control analysis does itself divide the metabolic core into three blocks: a catabolic block that supplies phosphorylation and reducing power, a biosynthetic block that makes the building blocks of macromolecular synthesis, and a block that makes and maintains cellular structure together with the gene and enzyme machinery, the three coupled through common intermediates and moiety-conserved cycles [11]. This tripartition is real, and it lies wholly inside what the TPB counts as one domain. It does not, however, individuate domains in the TPB’s sense, because its blocks are separated by substrate class and joined by shared intermediates, which is the asymmetric dependence just excluded, and because control analysis holds flux control to be a systemic property distributed across all steps rather than localized in any one of them [16], so that its blocks are not units that can fail independently. The two schemes answer different questions, and the TPB’s grouping is therefore not refuted by the existence of a finer metabolic partition.

The objection may nevertheless be right, and the framework should say what would show it. If a system is identified in which material transformation is selectively abolished while energy transduction remains intact, with a failure signature distinguishable from energy failure, and in which a four-domain model outperforms the three-domain model under matched parameterization, the TPB should be revised to four domains rather than defended. This is the most likely concrete instance of the general fourth-domain argument in Section 10.3, and it is the one a critic of the framework should pursue first. The asymmetry of scope between the domains is real; the claim is only that scope is not the criterion by which domains are individuated, and that non-substitutability is.

### 2.3. Assigning a Process to a Domain

Definitions broad enough to describe biology are broad enough to permit assignment after the fact, and the objection that receptors, transporters, translation and second messengers could each be placed in any of the three domains is correct as an objection to the definitions alone. The framework therefore does not rest on the definitions. It rests on a rule, stated here, that fixes assignment before the outcome is known and that can itself fail.

(i) Assignment is by primary causal role with respect to the specified emergent property, not by molecular identity. The question is never what a component is but what its selective removal does to the property under study. A receptor is assigned to Interface/Boundary when the property is selective admission of a signal, and to Information when the property is the fidelity of the downstream regulatory state. Both assignments are legitimate; what is prohibited is making them in the same analysis, or switching between them once the data are seen. The emergent property is registered under gate 2 in Figure 3 precisely so that assignment has a fixed referent.
(ii) Weighted membership is permitted, and must be declared in advance. A process may be assigned fractional memberships summing to unity across the three domains, and Section 3.3 shows how these weights enter the domain terms of the closure index as exponents in a weighted geometric mean, so that an indicator with divided membership contributes proportionally to more than one domain rather than being forced into one. Permitting weights without registering them would make the index unfalsifiable; registering them makes divided membership a declared modeling choice whose consequences are computed rather than a license to relabel.
(iii) A process that acts across all three domains is an integrator, not a domain, and is excluded from the domain terms. The operational test is that selective perturbation degrades more than one domain score by comparable magnitude, with the threshold for comparable pre-registered. Integrators enter the framework through the coupling terms, where their biological role actually lies, and not through the domain terms, which they would otherwise render statistically non-independent.

Worked example: AMP-activated protein kinase (AMPK). For the property maintenance of ATP concentration within the viable range under nutrient restriction, loss of AMPK degrades energetic integrity, and rule (i) would assign it predominant weight in Energy/Material Transformation. For the property coordination of catabolic and anabolic programs with nutrient availability, loss of AMPK degrades the correspondence between energetic state and transcriptional state without necessarily lowering either in isolation—coupling failure as defined in Section 7.2—and it belongs in the coupling term between Energy and Information rather than in any domain term. Because the same molecule receives materially different treatment under two legitimate property specifications, rule (iii) applies: at the cellular scale AMPK is declared an integrator; is excluded from I, E and B; and is used only in coupling estimation. Intracellular Ca^2+^ and mTORC1 are handled identically, and all three are treated as integrators in the cellular example in Section 4.2. The consequence for the worked example of Supplementary Section S2 is that ionized calcium may be used as an Interface indicator of membrane excitability and secretory competence, while Ca^2+^ as a second messenger may not be used as a domain indicator at all.

### 2.4. Fixing the System Boundary

The framework requires that the system boundary, scale and time window be fixed before analysis, and a critic is entitled to ask how, since a boundary chosen to produce a triad will produce one. Three constraints govern the choice. First, the boundary is selected by the emergent property and never by the domains: the investigator names the property to be explained—persistence of a bacterial population through nutrient limitation, maintenance of blood calcium within range through the first week of lactation, sustained remission of an autoimmune process—and the system is whatever must be intact for this property to obtain. This is settled before any domain is named, and the ordering of gates 1 and 2 in Figure 3 exists to enforce it. Second, the time window must be at least as long as the slowest coupling under study, since a coupling cannot be estimated from a single observation; this constraint alone often settles the question. Third, and decisively, the boundary must be held fixed under perturbation. Table 1 rejects mappings whose boundary changes to preserve a triad, and a boundary redrawn when a counterexample appears has failed regardless of how natural the redrawing seems.

Applied to the hard cases, the rule gives determinate and uncomfortable answers, which is the point. For a virus, if the property is persistence and evolvability of the viral lineage, the system is the infected cell together with the viral genome, and the virion is by construction a scaffolded rather than an autonomous system—a claim that is falsifiable, since a virion shown to maintain, transform and bound itself outside a host would refute it. This stipulation is not idiosyncratic; virology contains the same dispute. According to the virocell account the metabolically active infected cell is the living form of the virus and the virion is its dispersal stage, the equivalent of a spore or a seed, so that equating the number of viral particles with the number of viruses is, in Forterre’s comparison, like estimating the number of fish in the ocean by counting fish eggs [17]. If the property is only physical stability of the virion in the environment, no triad is claimed and none should be sought; the TPB is silent about systems whose persistence requires no self-maintenance. For a mammalian host and its microbiome, microbial metabolic contributions are external inputs when the property is organismal thermoregulation and internal to the system when the property is colonic barrier maintenance. Here too the boundary is contested by the field rather than left vague by the TPB. The holobiont position treats host and microbiome as a single integrated biomolecular network and a unit on which selection can act [18]. Its critics argue that such an association is better treated as an ecological community, whose members’ evolutionary interests diverge and whose partner fidelity is too low for the hologenome to behave as a unit of selection [19]. The TPB does not adjudicate this dispute. It requires only that the choice be made first, made explicit, and held fixed for the duration of the analysis. The organizational literature reaches the same conclusion from another direction, finding that levels of autonomy can come apart within a single organism, so that a multicellular formation may fail to qualify as an autonomous system although its constituent cells qualify [20]. Both analyses are legitimate, they may not be merged, and a result obtained under one boundary may not be reported as support under the other.

### 2.5. Autonomy Without Circularity

That a system lacking an internally sustained domain depends on a host or scaffold for this function is analytic rather than predictive, and the claim would be circular if autonomy were itself defined as possessing three internally sustained domains. The empirical content lies instead in a graded relation. The definition used in this article therefore makes no reference to the domains. A system is autonomous over a specified interval to the degree that it maintains its characteristic organization while the externally supplied inputs are restricted to those of low specificity: free energy, and small molecules and ions available in the ambient environment. Dependence on a high-specificity input that the system cannot itself produce—a macromolecule, a template, a catalyst, a preformed boundary, or an engineered laboratory condition—is a deduction from autonomy in proportion to the specificity of this input and the rapidity of failure on its withdrawal. Specificity itself requires an operational definition, since it now carries the weight the domains previously carried. It is defined here as inverse ambient availability: the specificity of an input is the reciprocal of the probability of encountering it at functional concentration in the system’s habitat in the absence of another organism producing it. Free energy, water, inorganic ions and small organic molecules of abiotic provenance therefore score low; polymers, templates, folded catalysts, preformed membranes and engineered culture conditions score high, because each requires a biological or laboratory producer. The measure is coarse, and two investigators may reasonably disagree about a borderline input such as a vitamin or a cofactor. What matters for the present argument is that they can disagree without either of them appealing to Information, Energy/Material Transformation or Interface/Boundary, which is the property that removes the circularity.

So defined, autonomy is a measurable continuum assessed by progressive withdrawal of specific external supports with measurement of persistence, reproduction and evolvability, and it says nothing about information, transformation or boundaries. Defining autonomy separately from closure is not a departure from the organizational tradition but an operational rendering of a distinction that tradition already insists upon. Moreno and Mossio hold that autonomy is inherently grounded in organizational closure and yet is not equivalent to it, reserving the term for closed organizations that are also regulated and agential, and classing systems that lack these further features as infra-biological [20]. The TPB requires the same separation for a reason of its own, since its central claim is a claim about what closure demands; what it adds is a coarse quantitative proxy where the organizational account supplies a conceptual criterion [20]. The TPB claim then becomes non-trivial: it predicts that autonomy measured in this way will correlate with the internalization of the three domains, and it is refuted if a system scoring high on withdrawal-resistant persistence is found to lack one of them. Section 9.5 states the experiment, and the reduced systems in Section 10.4—the enucleate erythrocyte, the reduced endosymbiont, the obligate intracellular parasite, the minimal synthetic cell—are graded by this independent measure rather than by the TPB’s own vocabulary. The mature erythrocyte is thereby classified as low autonomy not because it lacks a nucleus but because it fails within days of withdrawal of a continuously renewed extracellular supply and, under any supply regime, neither repairs nor reproduces, and it would count as a counterexample if it did not. Four senses of autonomy are worth separating here, because a system may hold one and lack another. Reproductive autonomy is the capacity to produce a further system of the same kind without an external template or machinery; metabolic autonomy is the capacity to regenerate the system’s own components from low-specificity inputs; informational autonomy is the capacity to maintain and deploy its own constraints without an externally supplied program; and boundary autonomy is the capacity to build and repair the barrier that establishes what counts as inside. The withdrawal measure defined above is deliberately indifferent to which of the four is missing, since scoring it by which domain is absent would reinstate the circularity this section exists to remove; the four are distinguished so that a result can be reported as a loss of a specific kind of autonomy rather than of autonomy in general.

Independent assignment and inter-rater agreement. These judgments are not automatic, and a rule that only its author can apply is not a rule. The framework therefore requires that domain assignment for any prospective test be performed independently by at least two investigators blind to outcome, that disagreements be resolved and documented before unblinding, that the criterion for acceptable inter-rater agreement be specified prospectively, and that agreement be assessed before consensus resolution and reported with the result. An assignment procedure that cannot achieve acceptable agreement in a given system is evidence that the domains are insufficiently objective for that system; this finding should be reported transparently. Section 3.4 records low inter-rater agreement as a condition under which the TPB should be rejected rather than repaired.

### 2.6. Criteria for a Genuine TPB Triad

A theory that seeks triads is vulnerable to confirmation bias unless its examples are selected by criteria independent of the desired number. The TPB therefore requires a prospective test. Before counting components, the investigator must specify the system boundary (Section 2.4), the emergent property to be explained, and the competing decompositions—which must include not only dyadic and tetradic alternatives but the rival tripartitions in Section 2.2, since a framework that outperforms two- and four-part models while performing no better than sensing–regulation–action has established nothing about these three domains. The three proposed domains must then satisfy the eight criteria set out in Table 1 and applied as a decision procedure in Figure 3. Non-substitutability, the criterion that performs most of the work, means that no amount of additional activity in the remaining domains can take over the function of a failed one: no degree of metabolic vigor substitutes for a lost regulatory program, and no degree of transcriptional competence substitutes for an absent energy supply. A distinction must be drawn here that the criterion does not make on its own. A domain that resists compensation and a domain that resists manipulation look alike in a failed experiment, and the difference has to be settled by design rather than by interpretation. Three requirements are therefore imposed on any non-substitutability claim. The perturbation must be titrated, so that a dose–response relationship between its magnitude and the loss of the emergent property is demonstrated rather than inferred from a single maximal intervention. The achieved perturbation must be verified by direct measurement of the domain score, so that a null result can be attributed either to genuine compensation or to a perturbation that did not take place. And the compensation test must be run in the opposite direction, by supplying supraphysiological activity in the remaining domains and showing that it does not restore the property. A domain that cannot be perturbed to a verified degree is declared experimentally intractable at registration and is excluded from the non-substitutability claim for the system, rather than being counted as non-substitutable by default. A list of three names, a historical taxonomy, or three correlated biomarkers is not sufficient.

### 2.7. Organizational Closure and Emergence

Organizational closure, introduced in Section 1 and set out in Box 1, provides the theoretical bridge between three functions and a living whole. In closure-of-constraints theory, components of an organism act as constraints on processes that produce and maintain the constraints themselves [2,3]. A membrane constrains transport while metabolism produces membrane constituents; enzymes constrain reaction pathways while genetic and translational systems regenerate enzymes; regulatory networks preserve operating conditions that allow the networks to continue. One distinction must be made explicitly, because the terms are often used interchangeably and are not equivalent. Causal feedback is not organizational closure. A two-node loop in which A influences B and B influences A is causally reciprocal, but it is organizationally closed only if A and B jointly produce and maintain the constraints that make their own operation possible; the formal usage of Montévil and Mossio requires the second condition and not merely the first [2,3], and biological regulation supplies many examples of the first without the second [21]. The TPB uses the term in this stronger sense, with one deliberate weakening that should be recorded: where the closure-of-constraints literature specifies closure over an explicit set of constraints and the processes they act upon, the TPB proposes a coarse-grained decomposition of this closure into three types of function, and whether the coarse-graining preserves what matters in the formal account is itself an open question rather than an assumption. The proposal is not that closure mathematically demands exactly three nodes in every graph. It is that autonomous biological closure repeatedly requires these three types of function.

Emergence is equally important. A triad is not justified merely because each component is necessary. The interaction must generate a property that cannot be attributed to any domain in isolation. Examples include autonomous persistence in a minimal cell, faithful translation, metabolic homeostasis, immune competence, and coordinated recovery from perturbation. Emergence is operationalized by comparing the predictive or functional performance of the complete system with systems in which one domain or a coupling is selectively impaired. The critical unit of analysis is therefore the coupled architecture, not the number alone.

Box 1 **Organizational closure—what it means, and what it does not.**

A system is organizationally closed when its parts make each other possible: each part helps maintain the conditions the others need in order to keep working, so that no single part sits upstream of all the rest [2,3].

**A worked example.** A bacterium’s membrane holds the cytoplasm concentrated enough for enzymes to work at useful rates. These enzymes synthesize the lipids that build and repair the membrane. The enzymes are made by ribosomes, built in turn from components the enzymes supply, following instructions in a genome that only enzymes can copy. Trace any of these dependencies far enough and it returns to where it started.

**A contrast.** A car factory produces cars but does not produce its own robots, refine its own steel, or train its own engineers; remove the external supply and it stops. An organism uses externally supplied matter and energy to produce, maintain and repair much of its own organization from within, which is why an organism can be damaged and repair itself while a factory cannot [5,6]. The contrast is one of degree over the timescale considered, not an absolute: a factory may recycle materials and maintain parts of its own production line, and an organism depends throughout on external matter, energy and ecological relationships.

**What closure does not mean.** It does not mean closed to matter or energy. Living systems are thermodynamically open and must remain so, since maintaining organization requires a continuous throughput of free energy. Closure is a statement about organization, not about exchange.

**Why it matters here.** Closure is what separates a genuine triad from any three things that happen to vary together. Three biomarkers rising and falling in step are not a triad; three functions that each maintain the conditions the other two require are. This is the difference the criteria in Table 1 and the cascade in Figure 3 are designed to detect, and it is why the TPB is a claim about coupling rather than about the number three.

**How closure fails.** In two ways that look different clinically. A domain can fall outside its viable range—energy failure during ischemia, a breached epithelial barrier, a lost regulatory program. Or the domains can each remain individually normal while the timing, magnitude or direction of their influence on one another becomes incoherent. The second kind is harder to detect with single-marker testing and is what the closure index of Box 2 is designed to capture. Section 7.1 develops this proposed sequence.

Box 2 **Interpreting the triadic closure index Θ(t).**

        $\Theta (t)={\left[I(t)E(t)B(t)\right]}^{1/3}\times {\left[\prod_{(i,j)\in \varepsilon }C_{\mathrm{ij}}(t)\right]}^{1/m}$

        *node integrity (geometric mean of three domains)*

      *× coupling integrity (geometric mean of the essential couplings ℰ)*

* *

**What the index is for**

Θ(*t*) is a proposed composite score summarizing domain integrity and agreement with a preregistered coupling pattern. Its idealized upper limit is 1; values approaching 0 indicate severe impairment under the model. A biological threshold for loss of closure requires independent validation.

**What each symbol means**

**I(t):** functional integrity of the Information domain, scaled 0–1: genome stability, chromatin and transcriptional competence, regulatory set-points, immune memory.

**E(t):** functional integrity of the Energy/Material Transformation domain: mitochondrial function, ATP availability, redox balance, biosynthetic and degradative capacity.

**B(t):** functional integrity of the Interface/Boundary domain: membrane and epithelial barrier function, transport, receptor-mediated sensing, selective exchange. Each of the three domain terms is a viability score and not a scaled biomarker concentration: values near the idealized upper limit of 1 denote operation near the viable reference state, and deficiency, excess or dysregulation reduce the score. This is required by the framework itself, since Section 7.2 recognizes domain hyperactivation as a mode of failure alongside domain deficiency.

**Cij(t):** the integrity of the coupling between domains i and j, also scaled 0–1, measured against a predefined viable reference pattern rather than as raw strength of statistical association. Strong association is not the same as healthy coordination: a coupling should score low if its direction, sign, gain, timing or proportionality of response is abnormal, even when the measured association is strong, and two markers may correlate tightly simply because a common pathological process drives both. This definition is what makes coupling failure—domains individually within range but incoherently related—detectable rather than invisible.

**m:** the number of couplings judged essential for the particular system under study. This must be specified in advance.

**1/3 and 1/m:** these exponents denote geometric means—the cube root of the product of the three domain terms, and the m-th root of the product of the m coupling terms. A geometric mean is used instead of an ordinary average for the reason set out below.

**How to read the two halves**

The first bracket asks whether the parts are working. The second asks whether they are working together. Θ is the product of the two, so a system scores highly only when both conditions hold. This is the arithmetic statement of a claim made verbally throughout this manuscript: a living system is not a collection of functioning parts but a set of functioning relationships among them.

**Why the terms are multiplied rather than added**

Table 2 sets out worked values for three domains, assuming for simplicity that all essential couplings share the same value.

The third row is the most instructive one. An additive score over the same three domain markers reports a system with one wholly absent function as 67% intact; the closure index reports it as failed. This is one arithmetic representation of non-substitutability: no amount of activity in the remaining domains can compensate for a function that is wholly absent. The equation encodes the property; it does not establish that biology possesses it, and Section 3.3 states how the aggregation exponent would be estimated rather than assumed. Note also how Θ behaves across the first three rows: halving one domain costs about a fifth of the index, whereas losing that domain entirely drives it to zero. The index therefore distinguishes reduced function from abolished function, which is the distinction that matters clinically. The fourth row is the case that conventional single-marker panels cannot detect at all: every individual measurement is normal, yet the system has lost half its coordination.

**What the index is not**

Θ is a modeling scaffold, not a fitted universal law. Its numerical value depends on which indicators are chosen to represent each domain and on how they are normalized, so values are comparable only within a consistent protocol and should not be compared across studies that made different choices. Different systems may require weighted terms, explicit time delays, indirect couplings or viable-set constraints. Most importantly, *m* and the identity of the essential couplings must be pre-specified. If they are selected after inspecting the outcome, *m* becomes a free parameter that can be tuned to fit, and the index loses its evidential value—this is the failure that gates 1 to 3 in Figure 3 are designed to prevent. Nor does the index settle the theoretical question: a Θ that behaves as intended shows that the instrument works, not that the triadic hypothesis is correct.

**In summary**

Θ is an attempt to express, as a single quantity, the claim that a system’s state depends on the integrity of its functions and on the integrity of the relationships between them.

**Table 2:** Behavior of the closure index under node failure and under coupling failure.

Scenario	I, E, B	Mean C_ij_	Θ	Additive Average
All domains and couplings intact	1, 1, 1	1.00	1.00	1.00
One domain partially impaired	0.5, 1, 1	1.00	0.79	0.83
One domain completely failed	0, 1, 1	1.00	0.00	0.67
Domains normal, coupling degraded	1, 1, 1	0.50	0.50	1.00

### 2.8. Antecedents and the Distinctive Contribution of the TPB

Tibor Gánti’s chemoton is the closest formal antecedent of the TPB. It models a minimal living unit as three stoichiometrically coupled autocatalytic subsystems—an open metabolic cycle, a self-replicating template polymer and a membrane—and its correspondence with Energy/Material Transformation, Information and Interface/Boundary is genuine [4,22]. Two qualifications matter. Gánti’s definition of the living state rests on five absolute criteria, not three, so the tripartition is his chemical implementation of these criteria rather than his thesis [4]; and the chemoton is a model of minimal chemical life that makes no claim about tissues, physiology, immunity or disease, best read as a heuristic platform for an exact theory of minimal life [23]. Autopoiesis [5], Rosen’s relational biology [6] and closure-of-constraints theory [2,3] are the other frameworks most often proposed as precedents. Wicken’s thermodynamic program [9,24,25] supplies the distinction between information and entropy adopted in Section 2.1 and the dyadic rival treated in Section 2.2, and the main criticism it attracted, that its thermodynamic considerations yielded no specific testable hypothesis [26], names the failure mode this article is built to avoid. The fuller historical record—the development of the chemoton and later analyses of its two-subsystem infra-biological variants [27,28], the two parts of Wicken’s work and their different reception, and a comparison of the TPB with the four antecedent frameworks on eight dimensions fixed in advance (Table S1)—is given in Supplementary Section S1.

The TPB advances a categorically broader, and correspondingly less secure, claim: that the same three-function architecture recurs across biological scales—molecular machinery, cellular organization, physiological regulation, immune defense, and integrated organismal response—and that its progressive loss constitutes a general model of pathogenesis. Four consequences follow that have no counterpart in chemoton theory. First, because the TPB must be applied to systems that were not stipulated in advance, it requires prospective admissibility criteria to distinguish a genuine triad from an arbitrary grouping at any scale (Table 1, Figure 3); this problem does not arise for a single defined minimal unit. Second, the TPB classifies modes of failure—deficiency, hyperactivation, hijack, bypass, coupling failure, compensatory exhaustion and collapse (Section 7.2)—converting an architectural proposal into a pathological vocabulary. Third, it supplies a quantitative closure index (Box 2) and a set of falsifiable predictions in network, perturbational, biomarker and therapeutic form (Section 9). Fourth, it makes clinical predictions, none of them tested: that the first-failing domain should be identifiable, that pre-specified multi-domain panels should outperform size-matched alternatives, and that durable therapeutic response should follow restoration of closure rather than the number of agents administered.

The relationship is therefore one of precedent rather than of anticipation, and it should be stated with care. Gánti formally modeled a triadic functional organization as a candidate sufficient architecture for minimal chemical life, with a rigor this paper does not attempt to match at this scale. He did not demonstrate it: the chemoton establishes formal sufficiency under stipulated chemical assumptions, no chemoton has been synthesized, and the claim that a tripartite organization suffices for life in any empirical sense remains open. This question was not posed within chemoton theory, and it can be answered only by the comparative, perturbational and clinical program set out in Section 9.

#### Formal Statement of the Triadic Principle of Biology

For a specified biological system and emergent function, stable self-regulation requires coordinated closure among three functionally distinguishable domains: (i) Information, which stores, interprets, and adaptively deploys biological constraints; (ii) Energy/Material Transformation, which performs the metabolic, chemical, and mechanical work of the system; and (iii) Interface/Boundary, which selectively mediates exchange, sensing, and protection between the system and its environment.

The domains may be realized by multiple components and may overlap materially. A system qualifies as a TPB triad only when the domains are non-substitutable for the defined function, reciprocally coupled, and jointly generate an emergent property that is impaired by domain-specific perturbation.

## 3. The Triadic Minimum: Mathematical Rationale and Limits

This section asks whether any reason independent of biology favors three. The answer is largely negative, and this section is arranged to make this explicit: Section 3.1 gives the one exact result and why it is not evidence, Section 3.2 shows that three bodies of formal reasoning do not support the framework either, and Section 3.3 and Section 3.4 carry the substantive content.

### 3.1. The Triplet Codon: An Exact Result of Limited Relevance

The one exact mathematical result available concerns the genetic code. Let *b* = 4 be the number of nucleotide symbols in the alphabet and let *k* = 20 be the number of standard amino acids. If at least one additional output is required for termination, the minimum fixed codon length *n* must satisfy *b^n^* ≥ *k* + 1. For *n* = 1, 4^1^ = 4; for *n* = 2, 4^2^ = 16; and for *n* = 3, 4^3^ = 64. Therefore, under these assumptions, *n*_min_ = ⌈log_4_(21)⌉ = 3. The classic frameshift experiments of Crick and colleagues provided the decisive early evidence that the code is read in non-overlapping triplets [29], although the full mechanistic account of decoding and reading-frame maintenance developed through substantially later work; the origin and evolutionary logic of the code remain actively disputed [30,31].

4^2^ < 21 ≤ 4^3^ ⇒ *n*_min_ = 3 (1)

This is a conditional proof about one historically realized coding problem, not a universal proof that biological architectures must have three nodes, and three qualifications keep it from being over-read. First, the termination requirement is not load-bearing, since 4^2^ = 16 is already less than 20. Second, and more importantly, the result concerns the minimum length of a code word whereas the TPB concerns the number of functional domains; these are different senses of three, and the inequality is therefore not evidence for the principle. Third, it explains why a triplet code suffices given twenty amino acids, not why the code is triplet: a two-nucleotide codon over a four-symbol alphabet provides 4^2^ = 16 possible codons, and several models of code origin treat the expansion to triplets as contingent—the code may be, in Crick’s phrase, a frozen accident [30,31]. The example is retained only because it is exact and was not arrived at by anyone looking for the number three.

### 3.2. What a Third Element Makes Possible, and What It Does Not Establish

Three bodies of formal reasoning—from graph theory, control theory and nonlinear dynamics—are sometimes invoked in support of triadic organization. None establishes the necessity of the TPB’s three functional domains. In graph theory, a two-node directed cycle can be strongly connected, so connectivity and functional differentiation do not require three nodes; a third node adds mediation, transitivity and the feed-forward loop, and three-node motifs are enriched in transcriptional and signaling networks [32,33,34]. But these are local wiring patterns among molecules, whereas the TPB coarse-grains biological work at a higher level, and transposing one to the other would be exactly the level confusion the framework must avoid; the two meet only in the function-preserving network test in Section 9.1, which is not a motif statistic. In control theory, Ashby’s law of requisite variety concerns the state variety a regulator needs, not a required number of components [35]. The applied literature on homeostatic control in physiology makes the same point: a regulated variable requires a sensor, a set-point and an effector, and how many components implement these roles is not fixed by the control problem [10]. In nonlinear dynamics, two-dimensional systems already show bistability and excitability while three-dimensional autonomous systems additionally permit chaos [36]; dimensionality is not the number of functional domains.

What they jointly establish is weaker than triadic necessity: a third element makes possible operations two cannot perform—mediation, sign-sensitive delay, persistence detection, separation of state from context—which biological regulation demonstrably requires. This is a reason to test a three-domain hypothesis, not an argument that three is necessary. The TPB’s own assertion owes nothing to these formalisms: that disturbances to information, transformation and interfacing are not generally reducible to one another, and that a system unable to correct these classes separately will lose autonomy in the sense described in Section 2.5. Whether three is the minimal sufficient decomposition is settled by intervention and model comparison.

### 3.3. The Conditional Triadic Minimum and the Operationalized Closure Index

A quantitative scaffold follows from the proposition. Let I(t), E(t) and B(t) be normalized functional integrity scores for the three domains and let C_ij_(t) be normalized coupling-integrity scores between them. The triadic closure index is the product of the geometric mean of the domain terms and the geometric mean of the couplings specified as essential:

Θ(t) = [ I(t) · E(t) · B(t) ]^(1/3) × [ ∏(i^j^)∈ε C_ij_(t) ]^(1/m) (2)

where ε is the pre-registered set of essential couplings and m = |ε|. Without these, Θ restates the definition of closure in symbols rather than converting it into a measurement, and the criticism that it does so would be correct.

#### Conditional Triadic Minimum Proposition

Let S be an autonomous biological system whose persistence requires three mutually non-reducible functions: FI (information storage and adaptive control), FE (energy and material transformation), and FB (selective boundary-mediated exchange). Assume that each function must remain independently controllable under perturbation and that no single functional domain can fully substitute for another. Then any minimal functional decomposition of S contains at least three domains. If the specified system boundary requires no additional irreducible function, the minimal number is exactly three.

The formal result is conditional because the substantive biological claim lies in the premises: whether FI, FE, and FB are jointly necessary, mutually non-reducible, and sufficient for the chosen system and emergent property. The TPB is therefore tested primarily by biology, not by relabeling a mathematical identity as biological proof.

**Computing the domain terms.** Each domain term is a viability score and not a scaled biomarker concentration, since Section 7.2 recognizes hyperactivation as a mode of failure alongside deficiency. For an indicator x with a pre-registered viable interval and reference value x*, define the range-referenced deviation d(x) = 2|x − x*|/(x_hi_ − x_lo_), so that d = 0 at the reference value and d = 1 at either limit of the viable interval; for one-sided indicators such as β-hydroxybutyrate, d(x) = (x − x*)/(x_hi_ − x*) truncated below at zero, and for asymmetric physiology the two half-ranges are scaled separately. Integrity is then s(x) = 1/(1 + exp[κ(d(x) − 1)]), a bounded monotone function returning approximately 1.00 at the reference value, exactly 0.5 at the limit of viability, and approaching 0 beyond it. The steepness κ is pre-registered; κ = 6 is used throughout this article and gives s = 0.998 at d = 0 and s = 0.002 at twice the viable half-range. Where a domain is represented by several indicators, the domain term is their weighted geometric mean, I(t) = [∏_k_ s_k_(t)^w_k_]^(1/∑w_k_), and the weights w_k_ are the declared domain memberships described in Section 2.3. An indicator assigned fractional membership to two domains therefore contributes to both terms in proportion to its declared weights, which is what permits weighted membership without making the domain terms arbitrary.

Computing the coupling terms. A coupling term scores the agreement of an observed cross-domain relationship with a pre-registered viable reference pattern, and not the raw strength of statistical association; this distinction is what makes coupling failure detectable rather than invisible, since two markers may correlate tightly simply because a common pathological process drives both. For domains i and j observed over a window at least as long as the slowest coupling, estimate the dependency as the partial correlation between the domain scores at the lag that maximizes it, conditioned on the third domain, namely, ${\hat{\ell }}_{\text{ij}}$ = argmax over ℓ ∈ L of |ρ(s_i_(t), s_j_(t − ℓ)|s_k_(t))|, with ${\hat{\rho }}_{\text{ij}}$ the signed partial correlation at that lag and L a pre-registered lag set, so that a reversal of sign registers as a large deviation from the reference rather than being absorbed by the maximization. This is the lagged conditional-independence estimator used in time-series causal discovery, and the procedures and limitations developed in the literature apply directly here, including the treatment of autocorrelation, unobserved common drivers, and the multiple testing induced by searching over a lag set [37,38]. The reference pattern must be estimated by the identical lag selection procedure in the reference cohort, and the inflation of $\left|\hat{\rho }\right|$ induced by selection over L reported. Coupling integrity is then the agreement of both the magnitude and the timing with the reference pattern (ρ*_ij_, ℓ*_ij_) obtained from a healthy reference cohort under the same protocol:

(3) $$C_{\mathrm{ij}}=\exp\left[-\alpha {\left(\left({\hat{\rho }}_{\mathrm{ij}}-\rho_{\mathrm{ij}}^{*}\right)/\sigma_{\rho }\right)}^{2}-\beta {\left(\left({\hat{l}}_{\mathrm{ij}}-l_{\mathrm{ij}}^{*}\right)/\sigma_{l}\right)}^{2}\right]$$

with *α* and *β* pre-registered tolerance parameters and *σ_ρ_*, *σ_ℓ_* the reference-cohort dispersions. Because the exponent is quadratic in the deviation and not monotone in $\hat{\rho }$, a coupling scores below 1 when it is weaker than reference, when its sign reverses, when its timing shifts, and—importantly—when it is stronger than the reference, which is how pathological over-coupling and loss of physiological independence are captured. Because the influence of *i* on *j* need not equal the influence of *j* on *i* in strength, timing or causal role, the two directions are estimated separately where the sampling design resolves them, and the bilateral term is their geometric mean C_ij_ = (C*_i→j_* · C*_j→i_*)^1/2^. Where sampling frequency cannot resolve direction, this must be declared and the undirected estimate used, with the limitation reported. Where the domain scores carry substantial measurement error, a state-space formulation that separates process variance from observation variance is preferable to correlating the raw scores, and the identifiability problems of this approach must be checked rather than assumed away [39].

Fixing ε and m before the outcome is known. If the essential coupling set were chosen after inspecting the data, m would become a free parameter tunable to fit and the index would lose all evidential value. The rule is therefore as follows. A coupling is essential if the emergent property cannot be maintained when that coupling alone is perturbed, judged from prior research or from pilot perturbation performed before the confirmatory data are collected, and the justification for each inclusion and each exclusion is recorded at registration. In the absence of such evidence the default is ε = all six directed couplings—the two directions of each of the three bilateral pairs—and m = 6; where the sampling design cannot resolve direction, the three bilateral terms are used instead and m = 3. This default is the conservative choice because it cannot be tuned to the outcome. It should be stated precisely rather than loosely: adding an inessential coupling whose integrity is below the geometric mean of the retained set lowers Θ, and one above it raises Θ, so the default is not uniformly attenuating. What it does guarantee is that the coupling set is fixed by rule rather than chosen after the outcome is seen, and any departure from it must be justified mechanically and recorded at registration. Any deviation from m = 6 must be declared and justified before unblinding, and reported with the result.

**Reference data, missing observations, and uncertainty.** Four further requirements complete the estimator, and each closes a place where an unstated choice would let the index be tuned after the fact. First, viable reference regions and the parameters of s(x) must be fixed from external biological knowledge or estimated in an independent reference sample, and never in the sample used to test the prediction; where one study must do both, the reference and test partitions are separated before any outcome is examined. Second, indicators within a domain must be checked for redundancy before aggregation, since two tightly correlated markers otherwise contribute twice and inflate the domain term. Third, completeness requirements and the handling of missing observations must be pre-registered: the pattern of missingness is reported rather than assumed ignorable, any imputation is estimated within the reference partition alone and carried through as multiple imputation so that its uncertainty propagates into the index, and complete-case and alternative-imputation analyses are reported as sensitivity checks. Fourth, every reported value of Θ and of its domain and coupling terms carries an uncertainty interval, because error in the reference regions, the indicators and the couplings all propagate into the aggregate; a point estimate without an interval is not interpretable. It follows that any threshold for loss of closure must be derived and validated in independent data and cannot be read off the equation. These are the reporting standards already established for prediction models [40,41], and the framework claims no exemption from them. One limit should be stated plainly. The selection of indicators, the normalization scheme, the identification of essential couplings, the choice of reference pattern and the handling of time lags are all discretionary decisions, and each can materially change the result. The index is therefore only as strong as the pre-registered choices beneath it. It is a test instrument for the hypothesis and not evidence that the hypothesis is correct.

**Figure 4:**
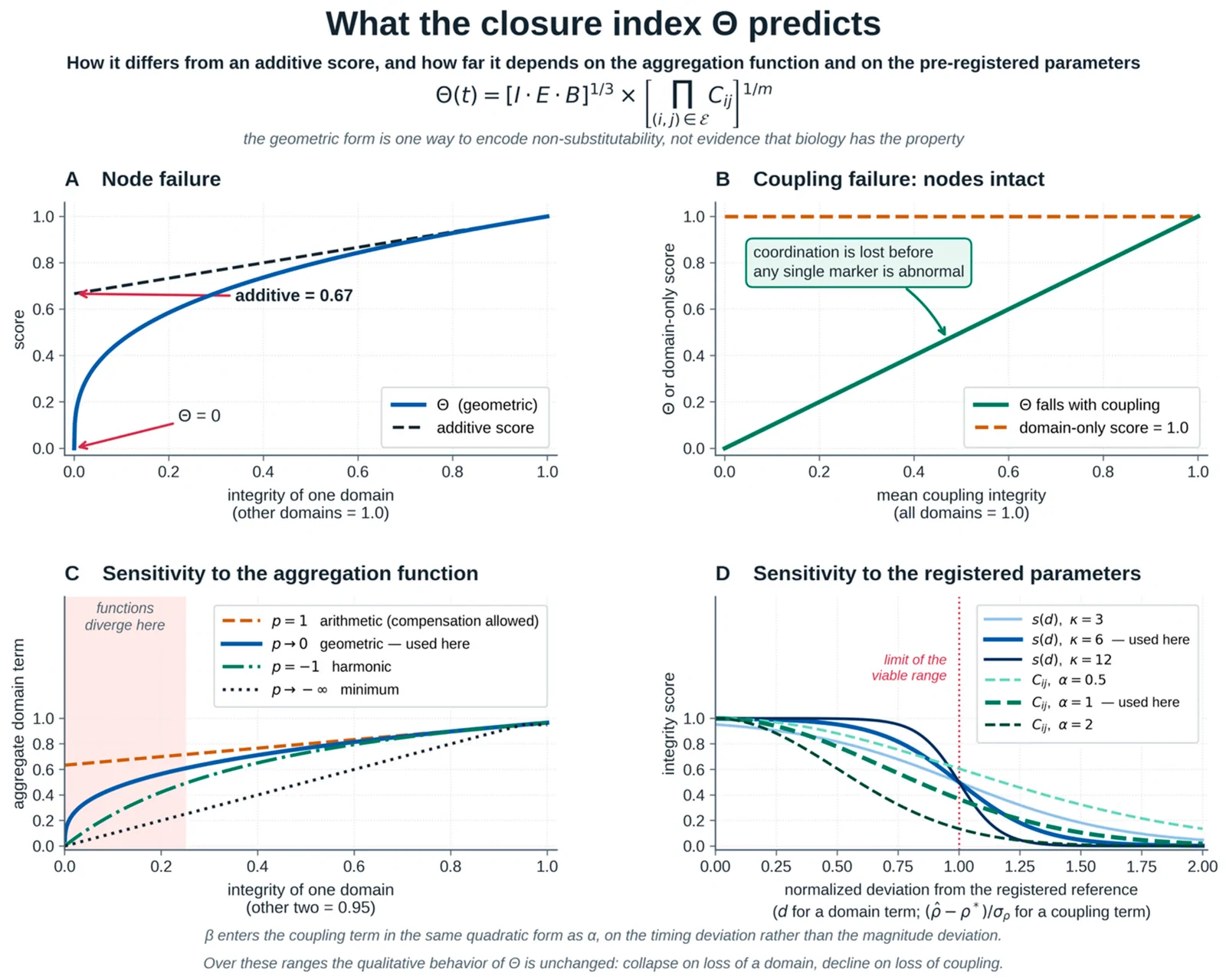
Behavior of the triadic closure index Θ, and how it differs from an additive multi-marker score. (**A**) Because the domain terms enter as a geometric mean, collapse of a single indispensable domain drives the index toward zero while an additive panel built from the same three markers still reads 0.67. This is one arithmetic representation of non-substitutability rather than evidence for it: the index cannot be rescued by compensatory activity elsewhere, but whether biology has the property is an empirical question addressed in Section 3.3. (**B**) Θ also declines when every domain is individually within its viable range but the couplings between them degrade. This dissociation is the sharpest testable claim generated by the framework, because it predicts that loss of cross-domain coordination should be detectable before any single marker crosses a conventional abnormality threshold; Supplementary Table S2 gives a worked numerical instance in the periparturient cow. (**C**) Sensitivity to the aggregation function. The same domain values are aggregated by the generalized mean M_p_ for *p* = 1 (arithmetic), p → 0 (geometric, used here), *p* = −1 (harmonic) and p → −∞ (minimum). The functions are nearly indistinguishable for mild, balanced deficits and diverge sharply as one domain approaches zero, which is where the framework’s substantive claim lies. TPB’s claim is *p* ≤ 0, not p = 0 specifically; the value of *p* is an empirical parameter to be estimated rather than a property the framework asserts. (**D**) Sensitivity to the parameters that must be registered in advance. The domain-integrity function s(d) = 1/(1 + exp[κ(d − 1)]) is drawn for κ = 3, 6 and 12, and the coupling-integrity function C_ij_ = exp[−αz^2^] for α = 0.5, 1 and 2, both against the normalized deviation from the registered reference. β enters the coupling term in the same quadratic form as α, acting on the timing deviation rather than the magnitude deviation, and behaves identically. Over these ranges the sharpness of the transitions changes but the qualitative behavior does not: a domain term still collapses beyond its viable range, and a coupling term still falls on either side of the reference. What these parameters control is how abruptly these transitions occur, which is why they are fixed at registration rather than fitted to the outcome. Θ is a modeling scaffold requiring parameterization against data, not a fitted law. In panel D, $\hat{\rho }$ is the estimated partial correlation, ρ^*^ is its viable reference value, and σ*_ρ_* is the dispersion estimated in the reference cohort. The superscript asterisk marks a reference value and does not denote statistical significance.

Why the terms are multiplied rather than added, and how sensitive the conclusion is to this choice. The geometric mean is a modeling decision and not a finding, and the assertion that it is what non-substitutability looks like written as arithmetic overstates the case: the equation encodes one mathematical representation of non-substitutability and does not establish that biology possesses the property. The honest form of the claim is comparative, and it is best expressed through the generalized mean M_p_, of which the arithmetic mean (*p* = 1), the geometric mean (p → 0), the harmonic mean (*p* = −1) and the minimum (p → −∞) are all special cases. Table 3 shows their behavior on the same domain values. For *p* > 0 the complete loss of one domain can be offset by activity in the others; for *p* ≤ 0 it cannot, because M_p_ falls to zero whenever any domain does. Partial trade-off between domains remains possible at every finite *p* and is eliminated only in the limit p → −∞, so the property that distinguishes *p* ≤ 0 is the non-compensability of complete loss rather than the absence of trade-off altogether. The TPB’s substantive claim is accordingly *p* ≤ 0 and not *p* = 0. The geometric mean is adopted here as the least committed member of this family: the minimum function encodes non-substitutability more strictly but discards all information about the non-limiting domains, and cannot distinguish a system with one halved domain from one in which all three are equally impaired, as the last two rows of Table 3 show. The value of *p* is an empirical parameter that should be estimated from data rather than asserted, and a fitted *p* reliably greater than zero would falsify the non-substitutability claim regardless of any other result. Figure 4 shows this behavior, together with the sensitivity of the domain term to the choice of aggregation function. Because s(x) is logistic it is bounded strictly between 0 and 1, so a domain term never attains exactly zero in practice; the absent-domain rows of Table 2 and Table 3 are limiting illustrations, and the discrimination between *p* ≤ 0 and *p* > 0 must therefore be made by estimating *p* rather than by observing a zero.

**A worked example.** Supplementary Section S2 gives a complete illustrative registration and computation of Θ for the periparturient dairy cow, with hypothetical values (Table S2). Ionized calcium represents Interface/Boundary, β-hydroxybutyrate and non-esterified fatty acids represent Energy/Material Transformation, and haptoglobin and serum amyloid A represent Information. The last assignment is the weakest and is declared weak in advance, because acute-phase proteins are hepatic and inflammatory outputs several steps downstream of the regulatory decisions they are used to index [42,43]. In the illustration, a cow that later develops disease has every indicator within its viable interval three days before calving; an additive score separates it from a cow that stays healthy by 0.03, whereas Θ separates them by 0.29 because the coupling term has already fallen. No value of Θ in this article is derived from measurement.

What Θ can and cannot compare. The index is not a cross-system metric and should not be presented as one. Its absolute value has no meaning independent of the indicator set, the viable intervals, κ, α, β and m fixed at registration, and two studies making different choices cannot be compared by their Θ values at all. This is a genuine limitation and it narrows the claim: Θ is a within-protocol trajectory measure, comparing a system against itself over time and individuals within a cohort measured under one protocol against one another. Reporting a bare Θ value across studies would be meaningless, and the framework does not propose it.

Three quantities derived from Θ are nevertheless comparable across systems, and they are what the framework actually predicts. The first is the ordering of change: whether the coupling term declines before the domain terms. The second is the lead time, expressed in units of the system’s own characteristic timescale, between the decline in Θ and the crossing of conventional thresholds by individual markers. The third is the comparative performance of Θ against a size-matched additive panel computed from the same indicators in the same data. Each of these is dimensionless and ordinal rather than absolute, and each can be replicated by a different group in a different system provided κ, α and β are matched or their influence is reported; a sensitivity analysis over these parameters must accompany any claim that the coupling term declined first. If the framework is supported in transition cows and in sepsis, it will have been supported by the concordance of these three quantities and not by any shared numerical value of Θ. A reader who regards a metric as useful only when it flattens differences for direct cross-system comparison should treat Θ as a poor metric and these three derived quantities as the framework’s actual measurable predictions.

### 3.4. Falsification Conditions

The TPB would be weakened or falsified in its strong form by any of six findings. The first is an autonomous, persistent biological system—autonomy assessed by the withdrawal criterion in Section 2.5 rather than by the TPB’s own vocabulary—that lacks one of the three proposed functions. The second is a reproducible two-domain system that achieves the same emergent property and perturbational robustness as the proposed triad, of which Wicken’s exploitation–homeostasis pair discussed in Section 2.2 is the most developed published candidate [13]. The third is a fourth function that is consistently irreducible across system boundaries and cannot be incorporated into the three domains, of which the separation of energy transduction from material synthesis discussed in Section 2.2 is the most likely concrete candidate. The fourth is failure of triadic models to outperform equally parameterized dyadic, tetradic or alternative-triadic alternatives, where the comparators must include at least sensing–regulation–action, supply–demand–control and the exploitation–homeostasis dyad. The fifth is inter-rater agreement on prospective domain assignment too low to support blind classification. The sixth is systematic inability to assign domains before outcomes are known. These conditions are not rhetorical safeguards. They define the research program, and the principal conditions appear in Section 9 with the design that would test each.

## 4. Molecular and Cellular Manifestations

### 4.1. Translation as Template–Adaptor–Catalytic Scaffold

Protein translation offers one of the clearest descriptive tripartitions in molecular biology. Messenger RNA provides the template specifying codon order; transfer RNA couples nucleotide recognition to amino acid delivery; and ribosomal RNA forms the catalytic and structural core that aligns substrates and catalyzes peptide bond formation [44,45,46]. Removing any one of these roles abolishes canonical translation [44]. The triad is embedded within a larger apparatus that includes aminoacyl-tRNA synthetases, ribosomal proteins, initiation and elongation factors, GTP, ATP, ions, and quality control systems. Therefore, mRNA, tRNA, and rRNA are not alone sufficient for modern protein synthesis. They are described here as a functional core implemented by a more complex machine; irreducibility in the TPB sense has not been tested. One caution follows directly, and it applies wherever a molecular example is mapped onto the framework. The mRNA–tRNA–rRNA triad and the Information–Energy–Interface triad are compatible decompositions but not identical ones, and no one-to-one correspondence between molecule and domain should be inferred. Ribosomal RNA is the clearest case: it provides the peptidyl transferase catalysis [45], assigned here to Energy/Material Transformation, while also forming the decoding environment in which codon–anticodon pairing is monitored [44], assigned here to Interface. A component contributing to more than one domain is expected under Section 2.1 and is not a defect of the mapping; treating translation as though each molecule occupied exactly one domain would be.

The central dogma can likewise be described as a common three-stage architecture—DNA, RNA, and protein—but it should not be mistaken for a proof of the TPB. Crick’s dogma concerns forbidden transfers of sequence information, not a requirement that every biological information system contain exactly three molecular species [47]. RNA genomes, reverse transcription, non-coding RNAs, and epigenetic regulation complicate any literal three-object reading. The TPB value of the central dogma is functional: stable storage, regulated interpretation, and executed molecular function are separated yet causally linked.

### 4.2. The Cellular Functional Triad

At the cellular scale, the most defensible TPB formulation is functional rather than organelle-exclusive. The Information domain is implemented by the genome, transcriptional regulation, and heritable cellular state. The Energy/Material domain is implemented by metabolic networks, redox systems, biosynthesis, degradation, and—where present—mitochondrial bioenergetics. The Interface/Boundary domain is implemented by the plasma membrane or cell envelope, transporters, receptors, extracellular matrix interactions, and trafficking systems. This formulation applies to prokaryotes and eukaryotes. In eukaryotes, nucleus, mitochondria, and plasma membrane are prominent hubs, but they are not the only essential structures. The endoplasmic reticulum, lysosomes, ribosomes, cytoskeleton, and other organelles are indispensable components that frequently bridge the domains.

The cellular mapping is consistent with extensive bidirectional communication, which is documented independently of the TPB and does not by itself establish the mapping. Nuclear genes encode most mitochondrial proteins, while mitochondrial energetic state, metabolites, reactive oxygen species, calcium, and stress pathways alter nuclear transcription and chromatin [8]. Mitochondria can form regulated contact sites with the nucleus during stress, concentrating retrograde signals [48]. Plasma membrane receptors activate kinase cascades that modify nuclear transcription and mitochondrial survival thresholds [49,50]. Conversely, nuclear programs determine receptor and transporter repertoires, and mitochondrial ATP sustains membrane ion gradients and transport. Calcium transfer at endoplasmic reticulum–mitochondrial contact sites couples extracellular demand to metabolism and cell fate [51,52].

AMP-activated protein kinase and mTOR illustrate cross-domain integration. AMPK senses energetic stress and coordinates catabolism, mitochondrial quality control, transcription, and nutrient transport [53]. mTOR integrates growth factors, amino acids, energy status, and biosynthetic capacity to coordinate growth [54]. Neither molecule is a separate fourth TPB domain. They are integrators whose biological meaning arises from acting across the three domains. The same principle applies to calcium, redox signals, and organelle contact sites [51,52].

**Figure 5:**
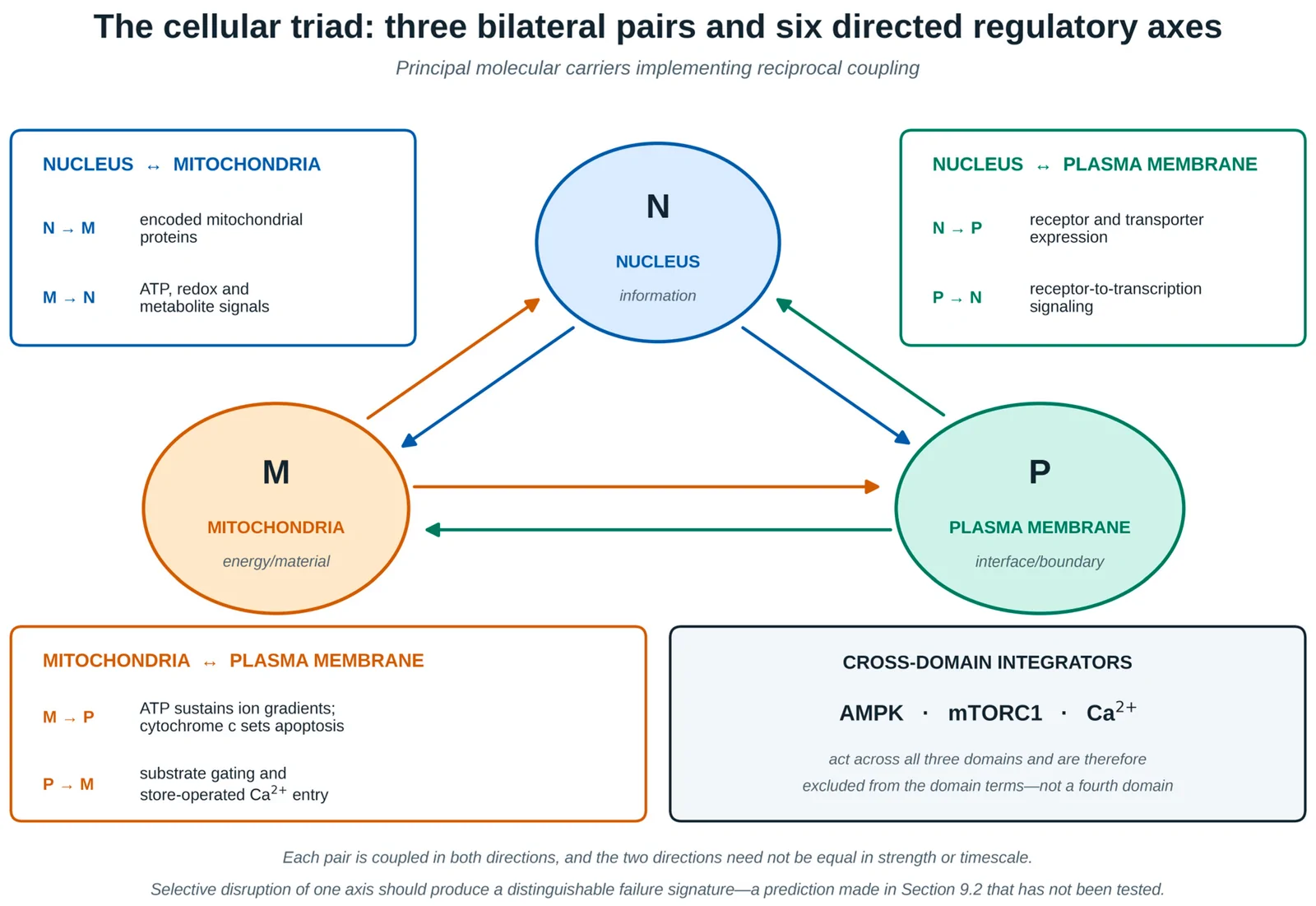
The cellular triad resolved into three bilateral pairs and six directed regulatory axes. In the eukaryotic cell illustrated, nucleus, mitochondria and plasma membrane are prominent hubs of Information, Energy/Material Transformation and Interface/Boundary respectively, and the principal molecular carrier of each direction is named. The figure is an instance and not a definition: these organelles are not the universal physical representatives of the three domains, the assignment does not hold for prokaryotes, which implement all three functions without either a nucleus or mitochondria, and in many cells a single organelle contributes substantially to more than one domain—the endoplasmic reticulum to boundary, synthesis and calcium signaling simultaneously. AMPK, mTORC1 and Ca^2+^ act across all three domains and are therefore integrators excluded from the domain terms under the rule in Section 2.3, entering the framework through the coupling terms instead. Selective disruption of each individual axis is predicted to produce a distinguishable failure signature (Section 9.2); this prediction is untested. N, M and P denote the nucleus, mitochondria and plasma membrane, respectively. Each arrowhead indicates the direction of influence; blue arrows originate at the nucleus, orange arrows at mitochondria, and green arrows at the plasma membrane. The six arrows form three reciprocal pairs, and the two directions within each pair need not have equal strength or operate on the same timescale.

These relationships are resolved into three bilateral pairs, and hence six directed couplings, in Figure 5, and Table 4 lists representative implementations of the three domains at other scales. Cellular homeostasis, fate decisions, and memory are proposed here to be emergent properties of this coupled architecture. A membrane can admit substrates but cannot alone determine whether their metabolism supports viability. Metabolic pathways can generate energy but cannot maintain their catalyst repertoire without information and biosynthesis. A genome can encode potential functions but cannot execute them without material transformation or a selectively maintained intracellular environment. The triad therefore describes a closure of dependency, not a hierarchy in which one node commands passive subordinates.

### 4.3. Minimal Cells and Synthetic Biology as Direct Tests

Minimal-cell research offers a direct testing ground. Gánti’s chemoton explicitly couples template replication, autocatalytic metabolism, and membrane production [4]. Synthetic-cell programs similarly seek systems capable of compartmentation, metabolism or energy transduction, information replication, growth, and division [62]. Bottom-up assembly has since produced vesicle systems that combine energy conversion with a defined boundary while still lacking heritable control, which is the pattern the TPB predicts for a two-domain construct [63]. The creation of a bacterium controlled by a minimized genome and the reconstruction of its essential metabolism demonstrate both the possibility and the difficulty of identifying minimal biological functions [64,65].

The TPB predicts that a genuinely autonomous synthetic cell cannot permanently outsource any of the three domains, where autonomy carries the independent meaning described in Section 2.5 and permanent outsourcing means continued dependence, over an interval exceeding several generations of the system’s own replication or turnover time, on a high-specificity external input the system cannot itself produce. A vesicle carrying a genome but no sustainable metabolism is not autonomous in this sense; a metabolically active compartment without heritable control cannot adapt across generations; a replicating template without a selective boundary depends on an external environment to supply and retain its organization. Systems that borrow a function from a host or laboratory are classified as dependent or scaffolded rather than as counterexamples, and this classification is not a definitional evasion because it is made by the withdrawal measurement in Section 2.5 rather than by inspecting which domain is missing. Recent progress in minimal and synthetic cells, including the genetic requirements for division in a genomically minimal cell [66], defines the natural test bed; the experiment is to withdraw external supports progressively and measure persistence, reproduction and evolvability [67].

## 5. Epigenetic and Developmental Manifestations

### 5.1. An Epigenetic Triad as a Working Functional Decomposition

Epigenetic regulation is often organized around DNA methylation, histone and chromatin modification, and regulatory non-coding RNA. DNA methylation can provide relatively stable, mitotically propagated repression or context-dependent regulation [55,56]. Histone modifications and chromatin remodeling alter accessibility and recruit regulatory complexes [57]. Non-coding RNAs provide sequence-specific targeting, scaffolding, and dynamic control [58,59]. These mechanisms interact bidirectionally: methylated DNA recruits chromatin regulators, histone states influence DNA methyltransferase targeting, and non-coding RNAs guide or modulate both.

This is a plausible triadic architecture, but it should be presented as a working decomposition rather than an exhaustive list of exactly three epigenetic mechanisms. Histone variants, ATP-dependent chromatin remodelers, nucleosome positioning, three-dimensional genome organization, RNA modification, and metabolite availability are also central. It would be circular to treat these simply as implementation layers distributed across memory, architecture and dynamic targeting, because a framework that absorbs every additional mechanism into one of its three categories can never be shown to have the wrong number of categories. They are therefore registered here as a rival decomposition with the same standing as the triadic one: a five-part account in which ATP-dependent chromatin remodeling and nucleosome positioning form an architectural layer, three-dimensional genome organization a spatial layer, RNA modification a post-transcriptional layer, and metabolite-dependent chromatin regulation a metabolic-input layer, alongside methylation and non-coding RNA. The two are separated by an experiment and not by definition. The comparison is made for a defined locus and a defined property: stability of expression through mitosis, speed of induction, or specificity of targeting. The triadic model is supported only if perturbing one of its three categories degrades the property more than perturbing any single layer of the five-part account under matched effect size. It is supported only if, in addition, the five-part account does not predict the perturbation outcomes at least as well under matched parameterization. Until this comparison is made, this section records a candidate decomposition and not a result, and Table 4 lists the epigenetic mapping as failing rather than as supported. The triadic assignment rests on a coarse-graining choice that has not been independently validated, and the five-part account may prove equally or more adequate under matched parameterization.

### 5.2. The Three Germ Layers and the Expansion of Animal Complexity

During gastrulation, triploblastic animals form ectoderm, mesoderm and endoderm. These contribute, respectively, to epidermal and neural structures; muscle, connective tissue and the circulatory system; and much of the epithelial lining of the digestive and respiratory systems [68,69]. The origin of the mesoderm was a major transition whose developmental and genetic basis remains debated, and the accompanying expansion of internal organization should not be described as a simple consequence of adding a third layer [68,70].

Triploblasty is a developmental pattern and not evidence that the three TPB domains are required; the two tripartitions are unrelated except by number. Not all animals are triploblastic, cnidarians possess contractile cells and complex tissue organization, and expression of “mesodermal” genes in the sea anemone complicates simple narratives [70]. Each germ layer contributes to several TPB functions, so a one-to-one mapping would be forced and none is proposed; the example is recorded in Table 4 as failing the criteria in Table 1.

## 6. Physiological, Evolutionary, and Ecological Manifestations

### 6.1. Canonical Endocrine Cascades

Several canonical vertebrate endocrine axes are organized as three-level cascades. In the hypothalamic–pituitary–adrenal axis, hypothalamic releasing signals regulate pituitary ACTH, which controls adrenal glucocorticoid production, with feedback to upstream levels [71]. Comparable three-level architectures occur in the thyroid [72] and gonadal [73] axes. These examples are tripartite in a descriptive sense: they separate integration, signal translation, and peripheral execution while maintaining feedback. The three levels are stages of one control cascade and do not correspond to the three TPB domains.

The claim must remain qualified: not every endocrine system has a hypothalamus–pituitary–target gland structure. Many endocrine and neuroendocrine circuits follow different architectures. The TPB therefore treats three-level axes as descriptive illustrations of tiered control, not as instances of an irreducible TPB triad and not as evidence that every hormone pathway has exactly three anatomical nodes.

### 6.2. Barrier, Innate, and Adaptive Immunity

Immune defense is conventionally described in three parts: physical and chemical barriers regulate access; innate immunity rapidly detects conserved danger patterns and executes inflammatory, phagocytic, complement, and antimicrobial responses [61]; adaptive immunity generates receptor diversification, clonal selection, specificity, and memory. The three are not independent “lines” acting in sequence. Barrier cells produce cytokines and antimicrobial molecules; innate antigen-presenting cells instruct adaptive responses [60]; antibodies and T cells alter innate effector activity [74] and barrier function [75]. Competent defense emerges from cross-regulation.

The TPB mapping is functional and is one possible coarse-graining rather than the natural decomposition of immune organization: barrier and sampling systems implement Interface, innate effector mechanisms much of Energy/Material Transformation, and adaptive memory Information. The middle assignment is the least self-evident and rests on evidence that innate effector phenotype is causally shaped by metabolic reprogramming rather than merely accompanied by it [76,77]. Each branch also contains all three functions internally, which is expected under the framework but is also what makes the mapping difficult to falsify; Table 4 records it as requiring the perturbational test in Section 9.2 before it can count as support. The assignment is provisional, and the recognition–effector–regulation decomposition listed in Table 4 may prove equally or more adequate under matched parameterization.

### 6.3. Sensor–Integrator–Effector Loops and the Tripartite Synapse

Physiological control often separates sensing, integration, and execution. The arterial baroreflex includes mechanosensory afferents, central integration, and autonomic effects on heart and vessels [78]. Reflexes should not be oversimplified as universally requiring a sensory neuron, interneuron, and motor neuron: the classical stretch reflex contains a monosynaptic afferent–motor connection. At the functional level, however, receptor, integrative comparison, and effector response remain distinguishable [10]. This is another example of why TPB counts functions rather than imposing a fixed anatomical diagram. Sensor–integrator–effector is, however, the rival tripartition in Section 2.2 and not the TPB decomposition, so these loops illustrate tripartite control and are not counted as TPB triads.

At synapses, the tripartite synapse concept treats astrocytes as active partners regulating transmitter clearance, ion balance and metabolic support [79,80]; the model is contested, and neither the extent nor the physiological significance of astrocytic signaling is settled [81]. It illustrates only that a mediating partner can turn a dyadic junction into a regulated local system, and is not evidence that synapses or control systems generally require three partners.

### 6.4. Evolution: Variation, Differential Fitness, and Heredity

The strongest evolutionary triad is not the taxonomic division of life but the classical conditions for evolution by natural selection: variation, differential survival or reproduction, and heredity [82]. These are conditions on a population over generations, not three mutually coupled functions maintaining closure within one system, and no correspondence with the domains is proposed. The example is included because its difference from the TPB clarifies what the TPB claims.

The three-domain classification of Bacteria, Archaea and Eukarya was transformative [83] but should not be mapped onto the domains: every taxonomic domain performs all three functions, and phylogenomics increasingly supports a two-domain tree [84,85,86]. The example is relinquished rather than defended.

## 7. Disease as Triadic Disruption and Recovery as Triadic Restoration

The periparturient dairy cow is used below as the principal worked system, not because the framework is about cattle but because it satisfies conditions that human chronic disease rarely does: the onset of the disturbance is known in advance to within days, the population is large and uniformly managed, samples can be taken repeatedly before clinical signs appear, and the demands on calcium regulation, energy metabolism and immune function all rise steeply within the same period. Few systems in mammalian biology present a scheduled, simultaneous three-domain perturbation in a population that can be followed prospectively, which is why it is the system in which the predictions made in Section 9 are most nearly achievable. Section 7.7 develops a human counterpart in sepsis.

### 7.1. From First-Failing Domain to System-Level Disease

The account in this section is a hypothesis-generating model and not a description validated by the literature. On this model, disease need not begin with simultaneous failure of all three domains. A pathogen may first breach an interface; a mutation may first alter information; ischemia may first constrain energetic transformation; an autoimmune program may first misclassify a signal; or a toxicant may disrupt several domains at once. Because the domains are coupled, the initiating disturbance changes the conditions experienced by the other two. Compensation may initially preserve function, but sustained perturbation can create positive feedback, loss of coordination, and eventual triadic decompensation.

**Figure 6:**
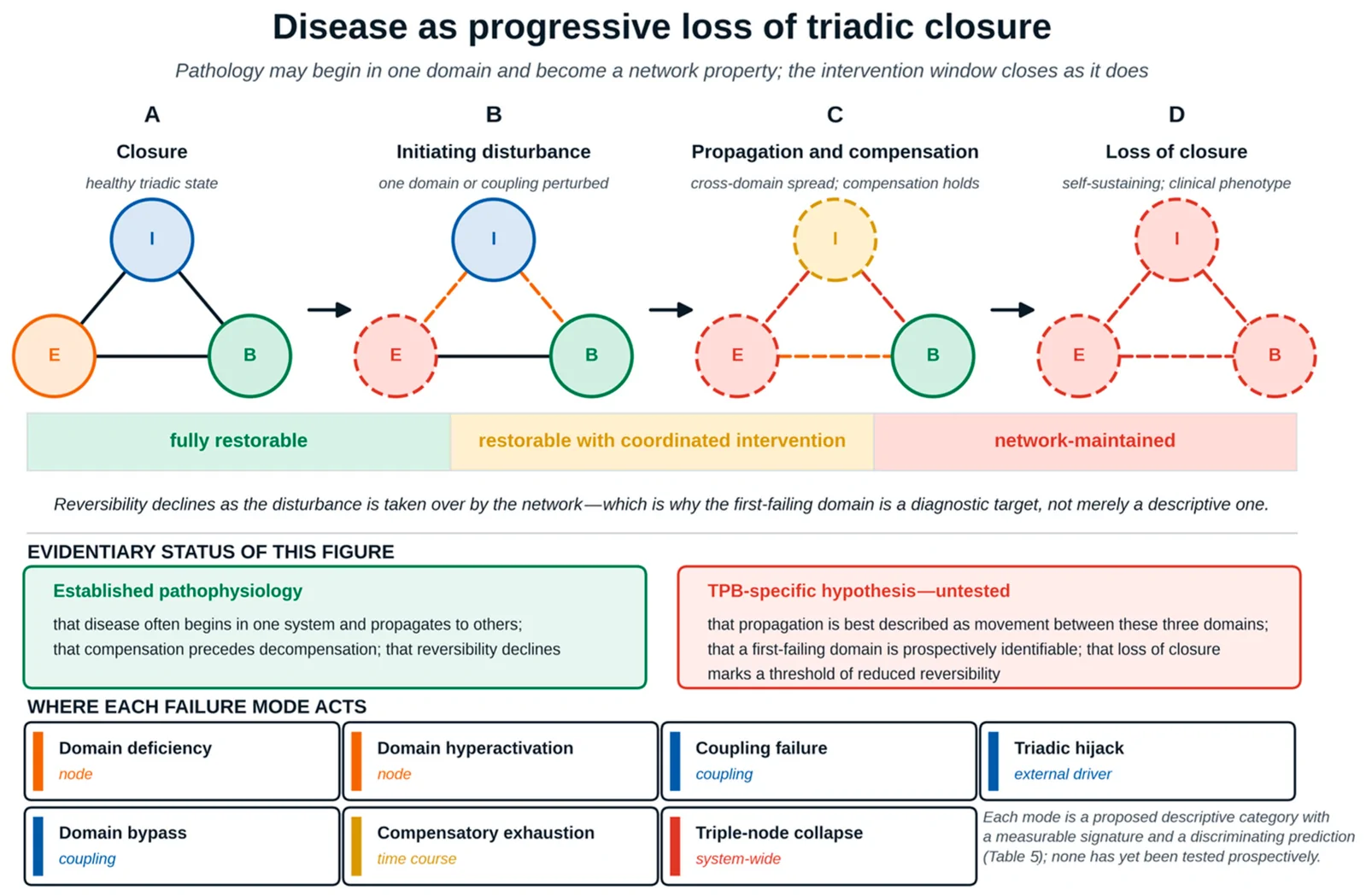
Disease as progressive loss of triadic closure. (**A**) The healthy state, with all domains and couplings intact. (**B**) An initiating disturbance in one domain or one coupling; early disease may be monodomain in origin. (**C**) Cross-domain propagation while compensation still preserves function. (**D**) Loss of closure, at which point the disturbance is maintained by the network and may persist after the initiating factor is removed. The colored gradient bar depicts a hypothesized decline in recoverability across the sequence; it is not a validated classification. Evidentiary status differs across the panels and should be read accordingly: that disease frequently begins in one system and propagates to others, and that compensation precedes decompensation, are established features of pathophysiology; that the propagation is best described as movement between three functional domains, that a first-failing domain is identifiable prospectively, and that loss of closure marks a threshold of reduced reversibility are TPB-specific hypotheses, none of which has been tested. The lower panel maps each failure mode in Table 5 to the element of the architecture on which it acts. Solid node outlines and connecting lines denote intact domain function and coupling. Dashed node outlines indicate compromised domain integrity; dashed connecting lines indicate impaired or dysregulated coupling. Amber denotes a compensated or transitional disturbance, and red denotes severe dysfunction in this schematic. The black arrows show the proposed progression between states, not measured time intervals or an inevitable course in every disease.

This proposed sequence would resolve an apparent contradiction between “disease as triadic disruption” and the clinically useful idea of a first-failing node. On this model, early disease may be monodomain in origin but triadic in propagation. Advanced disease may be maintained by the network even after the initiating factor is removed. The hypothesized temporal sequence is therefore initiating node or coupling disturbance → compensation → cross-domain propagation → loss of closure → clinical phenotype. Figure 6 summarizes the model.

### 7.2. A Taxonomy of Triadic Pathology

Table 5 summarizes the proposed modes of pathological change within the TPB.

The proposed failure modes motivate three corollaries concerning pathogenesis, diagnosis and treatment. Each remains a prediction requiring prospective testing.

#### Three Pathological Corollaries of TPB

**Triadic Pathogenesis Corollary:** In a system organized by three mutually dependent functional domains, disturbance of one domain changes the regulatory conditions experienced by the other two. Clinical disease is proposed to emerge when compensation no longer preserves closure within the viable range.

**Triadic Diagnostic Corollary:** The state of a triadic system is predicted to be represented more completely by measurements from all three domains and their couplings than by an isolated marker, provided that the multi-domain model outperforms size-matched alternatives in independent validation.

**Triadic Therapeutic Corollary:** Recovery is predicted to require restoration of the functions and relationships necessary for closure. This may be achieved by one upstream intervention, one pleiotropic intervention, sequential correction, or coordinated combination therapy; it does not require exactly three drugs.

### 7.3. The Transition Dairy Cow as a Worked Disease Application

The transition dairy cow provides a biologically rich test because calcium regulation, energy metabolism, immunity, endocrine adaptation, and tissue demands change rapidly around parturition [87,88]. The system is used here as a candidate application of the TPB and not as evidence for it, and the physiological relationships on which the application draws are independently documented: peripartal calcium homeostasis and its disorders [87], the coupling of metabolic state to neutrophil and broader immune function in transition cows [89,90], the influence of immune activation on transition cow metabolism and performance as evaluated by an independent group [91], and the role of the acute-phase response as a regulated systemic output rather than a direct measure of any single process [42,43]. Within the TPB, calcium dynamics are evaluated for an Interface role because Ca^2+^ mediates the coupling of extracellular signals to contraction, secretion and immune-cell activation at membranes; the caution expressed in Section 2.3 applies with particular force here, since Ca^2+^ acting as an intracellular second messenger is an integrator across all three domains and is excluded from the domain terms in this capacity. Two further objections to this assignment should be recorded, because both are reasonable. The quantity registered is ionized calcium in blood, an extracellular-compartment variable set by transepithelial absorption, renal handling and bone–blood exchange, which is why it is read here as an interface variable rather than as a measure of intracellular signaling; this reading is a choice about which process the indicator tracks, not a claim about calcium biology in general. Maintaining the gradient is nonetheless ATP-dependent, so the assignment amounts to a claim that boundary-level exchange rather than cellular energetics is rate-limiting for the specified property in this system. The claim is testable and is registered as such: if variation in ionized calcium tracks the energy indicators more closely than the barrier and secretory indicators over the observation window, the assignment fails and the registration records it as a failure rather than reassigning the indicator after the fact. Ketone and lipid metabolism are evaluated for the Energy domain, while inflammatory sensing, cytokine programs, and acute-phase coordination are evaluated for Information. These three functional mappings—of calcium, of ketones and lipids, and of inflammatory markers—are the author’s working assignments for a prospective test. The cited literature documents the physiology; it does not establish the mappings.

A triadic reading of this system is not justified by the presence of three biomarkers alone. Its significance depends on causal coupling: inflammatory mediators can alter calcium regulatory pathways [91]; calcium availability changes immune-cell signaling and muscle function [87,89]; negative energy balance and ketonemia alter inflammatory and antimicrobial activity [89,90]; and inflammatory responses redirect nutrient use [91]. Peripartal calcium status, energetic profile, and neutrophil function are empirically associated [89], while cows developing ketosis show pre- and postpartum alterations in innate immunity and metabolism [92]. These associations are established; the claim that they constitute a triadic architecture is not, and the TPB prediction under test is the narrower one that changes in cross-domain coordination precede conventional disease thresholds.

### 7.4. The Keto–Inflammatory Network

The Keto–Inflammatory Network, also proposed by the present author [93], extends ketone biology beyond the historical view of ketonemia as an inherently pathological endpoint. The underlying biology is independently established and should be cited as such: β-hydroxybutyrate is a respiratory fuel, a signaling metabolite acting at cell-surface receptors, an inhibitor of class I histone deacetylases and a substrate for lysine β-hydroxybutyrylation, and an immunomodulator [94,95]. It can inhibit NOD-like receptor family pyrin domain-containing 3 (NLRP3) inflammasome activation under specified conditions [96], while mitochondrial metabolic state is itself a determinant of immune-cell phenotype [76]. Its effects are concentration-dependent, tissue-specific and context-dependent, and severe metabolic disturbance can coexist with oxidative stress, immune dysfunction and inflammation [94,95]. The TPB offers a way of framing this context dependence—distinguishing adaptive ketone signaling from pathological loss of closure by reference to concentration, temporal context, tissue state, redox environment, and coupling to immune and calcium networks—but the framing is a hypothesis about how to organize these observations, not an additional finding.

### 7.5. The Lameness Triad and the Susceptibility–Resilience Gate

The Lameness Triad, a further proposal of the present author [97], reframes dairy cow lameness as the convergence of three systemic processes, namely, sustained endotoxemic pressure, endothelial glycocalyx degradation, and metabolic triage, the last being a reading of the homeorhetic partitioning of nutrients away from peripheral maintenance in early lactation, which was described long before the TPB [98]. Each component draws on independently established studies—the structure and barrier function of the endothelial glycocalyx and its degradation under inflammatory and endotoxemic conditions [99,100], the established pathogenesis of sole ulcer and white line disease [101], and the systemic and inflammatory antecedents of hoof lesions [102]—but their combination into a single triad is proposed rather than demonstrated. The Susceptibility–Resilience Gate specifies that clinical disease emerges when the combined triadic burden, amplified by cow-specific susceptibility, exceeds resilience capacity [97]. This formulation is more precise than an undifferentiated structural–metabolic–inflammatory grouping because it specifies a causal sequence that would, if correct, account for why many cows remain sound under shared husbandry conditions; the sequence itself is untested.

(Endotoxemic pressure × Glycocalyx degradation × Metabolic triage) × Susceptibility > Resilience(4)

The expression above is a conceptual statement of this gate and not a validated model; it has not been fitted to data, its terms are not currently measured on a common scale, and the multiplicative form is a hypothesis about non-substitutability among the three burdens rather than a finding. To become testable, endotoxemic pressure would require a circulating or hepatic marker of endotoxin exposure integrated over time; glycocalyx degradation a shed-constituent assay such as circulating syndecan-1 or hyaluronan [99,100]; metabolic triage an index of nutrient partitioning away from peripheral tissue maintenance; susceptibility a composite of prior lesion history, parity, claw conformation and genomic risk; and resilience a measured rate of recovery from a standardized challenge. Longitudinal studies showing inflammatory and metabolic changes before clinical lameness provide a foundation for prospective testing [102], but they establish predictive association and not that the proposed triad is causally organized. The TPB predicts that the highest-performing early-warning model will not necessarily be the largest omics model: a parsimonious panel spanning the three causal domains, combined with lameness history and measures of resilience, should be compared against single-domain, randomly selected, and unconstrained machine learning models under the reporting standards for prediction model development and validation [40,41].

### 7.6. Prion Collapse and Endotoxin Hijack as Contrasting Applications

A companion application distinguishes prion propagation from chronic endotoxin-driven neuroinflammation. Prion biology involves protein conformation templating without sequence change [103], which bypasses one specific control route rather than information in general: conformation itself carries heritable information, and the label is useful only under this restriction. Chronic lipopolysaccharide exposure instead activates encoded receptors, inflammatory transcription and blood–brain barrier disruption [104]. In the vocabulary of Table 5, prion propagation would be classified as beginning as domain bypass and progressing to triple-node collapse as templating becomes self-sustaining, so the proposed contrast, which is interpretive and untested, is bypass-to-collapse versus triadic hijack: one corrupts an internal control route, the other overdrives intact host machinery from an interface trigger.

The terminology earns its place only if it improves prediction, and Table 5 attaches a measurable signature and a discriminating prediction to each mode. NLRP3 activation in amyloid and tau pathology shows that interface-derived inflammatory signals couple to degenerative protein networks [105,106]; these findings establish inflammatory mechanisms in neurodegeneration but not a TPB-specific interface-to-information-to-energy pathway, which remains an interpretive layer requiring direct comparison against established causal and network models [107,108].

### 7.7. Complex Human Disease and the Limits of Monotherapy

Type 2 diabetes and cancer show why the therapeutic corollary must be stated carefully. Insulin resistance involves receptor signaling, hepatic and muscle metabolism, mitochondrial and lipid handling and transcriptional adaptation [109]; cancer involves genetic and epigenetic alteration, metabolic rewiring, immune and stromal interfaces and evolutionary resistance [110,111]. These establish complexity, not that either disease is best represented as triadic, and TPB is offered as a candidate coarse-graining for prospective comparison rather than a superior description. Neither is cured by labeling three nodes, and a single upstream or pleiotropic intervention may suffice; the testable claim is that durable control should correlate with restoration of the critical functions and couplings, however many agents achieve it.

Box 3 presents sepsis as a candidate human instance of triadic disruption.

Box 3 **Sepsis as a candidate human instance of triadic disruption.**

**Why this example**. The applications developed in this section are veterinary, for reasons of experimental access set out above. Sepsis is offered as a short human counterpart because failure of all three domains and of their coordination is already well described in the clinical literature, so a reader can judge the framework against knowledge they already possess. It is an illustrative hypothesis and not evidence that the TPB has been confirmed.

Interface/Boundary. Epithelial and endothelial barrier disruption, degradation of the endothelial glycocalyx with shedding of syndecan-1 and hyaluronan, increased vascular permeability and loss of selective exchange between blood and tissue [99,100].

**Information**. Dysregulated pathogen and danger sensing, unrestrained inflammatory transcription, failure of the resolution programs that normally terminate the response, and subsequent immune reprogramming in survivors [61,77,112].

**Energy/Material Transformation**. Mitochondrial dysfunction, a shift toward aerobic glycolysis in immune and parenchymal cells, impaired oxygen utilization despite adequate delivery, and consequent tissue energetic failure [76,77,113].

**The emergent failure**. What defines sepsis clinically is not the failure of any one of these but the loss of coordinated homeostasis across organ systems, together with the observation that the syndrome may persist after the initiating infection has been controlled, a course now described as a distinct chronic critical illness phenotype [114]. In the vocabulary of Section 7.2 this would be read as coupling failure progressing toward loss of closure; this reading is interpretive and has not been tested. The clinical corollary must be stated carefully: sepsis management depends on source control, timely antimicrobial therapy, hemodynamic support and organ support acting together, and the claim here is not that source control is unimportant but that source control alone does not reliably reverse established organ dysfunction, whose persistence and reversibility vary substantially across patients and time points.

**What would have to be shown**. The framework predicts that a pre-specified panel spanning barrier integrity, inflammatory regulation and cellular energetics should outperform size-matched single-domain, dual-domain and rival tripartition panels in independent validation under established reporting standards [40,41], and that measures of cross-domain coordination should deteriorate before individual markers cross conventional thresholds, adding information beyond existing dynamical early-warning approaches [115,116]. Neither has been tested. Sepsis is a harder test system than the periparturient cow precisely because its onset is rarely observed prospectively, which is why the cow rather than the patient carries the primary predictions in Section 9.

## 8. Relationship with Systems Biology and Stratodynamics

The TPB does not compete with systems biology. Systems biology provides the measurement, modeling, and perturbation apparatus needed to test the TPB: multi-omics, causal networks, dynamic systems, control analysis, single-cell resolution, and longitudinal phenotyping. The TPB contributes a candidate theoretical layer by specifying a coarse-grained architecture to be compared with alternatives. Network medicine has already shown that disease phenotypes emerge from interacting modules rather than isolated genes [107]. The TPB asks whether many such modules can be organized prospectively into three indispensable functional domains.

**Figure 7:**
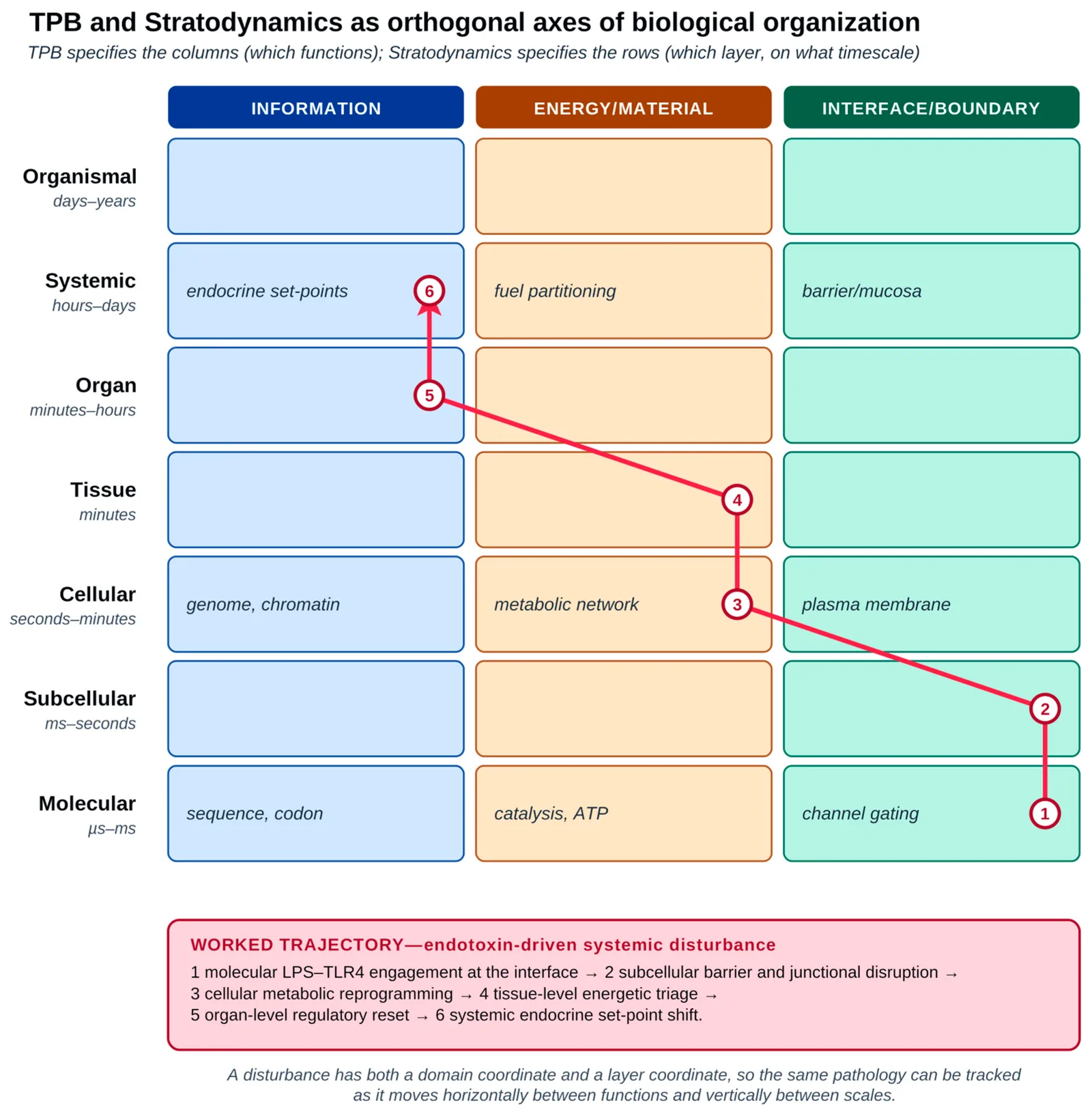
TPB and Stratodynamics as orthogonal axes of biological organization. TPB specifies the columns—which functional domains constitute the triadic core of a defined system. Stratodynamics specifies the rows—which layer of organization is involved and on what timescale it responds. A pathological process therefore has both a domain coordinate and a layer coordinate. Numbers 1–6 identify successive proposed stages in the illustrative endotoxin-driven trajectory: (1) molecular LPS–TLR4 engagement at the interface; (2) subcellular barrier and junctional disruption; (3) cellular metabolic reprogramming; (4) tissue-level energetic triage; (5) an organ-level regulatory reset; and (6) a systemic endocrine set-point shift. Stages 1–2 occupy Interface/Boundary, stages 3–4 Energy/Material Transformation, and stages 5–6 Information. The red arrows indicate hypothesized propagation between these domain–layer coordinates. The numbers are sequence labels, not measured values; the row timescales are schematic characteristic timescales, not measured delays for this trajectory. The falsifiable content is that the domain assignment of a given molecular event must remain stable as the process moves between layers; if the same event must be reassigned to a different domain at each layer in order to preserve the scheme, the cross-scale claim fails, and Section 9 records this as a falsification condition.

The TPB and Stratodynamics address different dimensions of organization. The TPB asks what functional architecture closure requires within a specified system. Stratodynamics, proposed by the present author and published in peer-reviewed form in this journal [117], holds that biological response is organized into seven functional layers with characteristic response times rather than along a continuum. That the framework itself has been published says nothing about the relationship proposed here between the two, which has not been tested and is a conjecture rather than an established distinction—the TPB defining a horizontal architecture of function, Stratodynamics a vertical and temporal coupling across layers. The general problem it addresses, that causation is not confined to a privileged level, is independently well established [118,119], and formal treatments of hierarchical functional organization long predate both frameworks [120], and readers should weight the relevant literature when assessing whether the cross-scale claim in Figure 7 is coherent.

## 9. Falsifiable Predictions and a Research Program

### 9.1. Network Enrichment Prediction

Curated biological networks can be annotated prospectively by function and system boundary. A closed functional triad is defined for this test by three requirements. It is a set of three nodes, one carrying predominant declared membership in each domain under the rule in Section 2.3. The nodes are connected by directed edges such that each domain is reachable from each other domain within a pre-specified path length, with indirect paths permitted only up to this length and declared in advance. And—the requirement that does the real work—each domain’s node must participate in at least one path that regenerates or maintains a constraint on which another domain’s node depends, these maintenance relations being declared at registration. The second condition is not optional. Mutual reachability alone is strong connectivity, which Section 2.7 distinguishes explicitly from organizational closure, so enrichment of merely reachable triads would leave the framework’s central claim untested. The TPB predicts that such triads will be enriched at control points governing persistence, homeostasis, differentiation, or recovery. The null model is the crux of the test and degree-preserving randomization is insufficient by itself [121,122], because function, subcellular localization, evolutionary conservation, expression level, network density and ascertainment bias all structure curated networks and any of them could generate apparent triadic enrichment. The specified comparison therefore uses a nested series of nulls: degree-preserving, then degree- and function-preserving, then additionally localization- and conservation-preserving, then additionally matched on study-level ascertainment. Enrichment must survive the full series. Domain labels are fixed before the network is examined and may not be permuted to maximize enrichment; the pre-registered label assignment is itself an object of the inter-rater check in Section 2.3, and candidate motifs surviving the nulls are then validated experimentally.

### 9.2. Perturbational Irreducibility Prediction

Selective perturbation of each domain should produce distinguishable failure signatures, while combined loss of essential couplings should impair the emergent property more than expected from independent effects. Experiments should measure recovery time, variance around the viable state, hysteresis, persistence under a second challenge, and capacity to transition adaptively. Weighted membership changes what a signature is predicted to look like, and the prediction must be adjusted rather than left in single-domain form. Where the perturbed component carries fractional membership under Section 2.3, the expected degradation of each domain score is proportional to the declared weights, and both magnitudes are stated at registration; a component with divided membership therefore yields a weaker test than one with predominant membership in a single domain, and the analysis plan must name which components carry the confirmatory weight. A perturbation whose observed effect on the two domain scores departs from the declared weight ratio by more than a pre-registered tolerance counts against the assignment, not merely against the perturbation. Importantly, the comparison must include a two-domain architecture with matched parameter count, a four-domain architecture that separates a proposed fourth function, and the rival tripartitions and the exploitation–homeostasis dyad in Section 2.2. Because these architectures are not nested within one another, model selection here is a non-nested problem and should be treated with the procedures developed for this case, in which candidate models are compared by their relative closeness to the unknown data-generating process and the possibility that neither is correctly specified is retained rather than assumed away [123]. The TPB is supported only if three provides the best explanatory balance of sufficiency, minimality, and robustness. One inference must be blocked explicitly: superior prediction by a triadic panel would not by itself establish a three-domain causal architecture, since a panel may predict well for reasons unrelated to the decomposition that generated it, and a correct architecture need not predict better than a well-chosen alternative. The architectural claim is carried by the perturbational tests of this section, not by predictive performance.

### 9.3. Biomarker Prediction

For complex disease, a pre-specified multi-domain biomarker model should outperform any single marker and all size-matched dual-domain models in independent validation, and should also be compared with randomly selected three-marker panels, with panels drawn from the rival tripartitions in Section 2.2, and with an unconstrained multivariable model. Comparison must follow established standards for prediction model development and external validation, reporting discrimination, calibration and clinical utility rather than discrimination alone, since a model may improve area under the curve while remaining poorly calibrated and clinically useless [40,41]. Two further constraints are adopted from the literature. The sample size must meet the minimum required for the number of candidate predictors rather than being fixed by convenience [124], and the comparison must include penalized logistic regression, since flexible machine learning models have not generally outperformed it for clinical prediction at these sample sizes [125]. In transition cows, one possible test combines an ionized-calcium indicator, a ketone or energetic indicator, and an inflammatory indicator, computed as in Section 3.3. The strongest TPB result would not be a higher area under the curve. It would be that loss of cross-marker coordination precedes abnormality of the individual markers and predicts disease across herds and management systems—a claim that must additionally be shown to add information beyond established dynamical early-warning approaches to critical transitions in disease, which make a superficially similar prediction from different premises [115,116].

### 9.4. Therapeutic Restoration Prediction

In established self-sustaining disease, interventions that restore the initiating domain but leave pathological coupling intact should show incomplete or transient benefit, whereas interventions that restore closure—through one upstream therapy or coordinated treatment—should produce more durable recovery. This prediction is circular unless restoration of closure is defined independently of the outcome it is used to predict, and the definition is therefore stipulated here. Restoration of closure is the return of the coupling terms C_ij_ to their pre-registered viable reference pattern, measured from indicators fixed at registration, at a time point specified in advance and strictly earlier than the outcome assessment. It is not defined by clinical improvement, and no variable used to define the outcome may enter the coupling estimate. Under this stipulation coupling restoration functions as a mechanistic mediator and is analyzed as such [126]: the design is factorial, the mediation is tested formally, and the framework is supported only if the intervention effect on durable recovery is substantially mediated by measured coupling restoration and not by domain term recovery alone. If coupling restoration were instead treated as a surrogate endpoint, the prediction would become untestable, and the TPB does not propose it as one. Because the prediction is also a claim about which individuals benefit, any subgroup analysis follows the standards for predictive approaches to treatment effect heterogeneity, with the risk or benefit model specified before the trial rather than derived from it [127]. The prediction is not that combination therapy is always superior, since multi-target action may also be achieved within a single agent [128] and unnecessary polytherapy carries its own risks. It is that therapeutic success follows restoration of the functions and relationships that maintain the viable state.

### 9.5. Minimal-Life and Dependency Prediction

Synthetic cells, obligate parasites, endosymbionts, and viruses provide a continuum of autonomy. The TPB predicts that systems lacking an internally sustained domain will depend on a host or experimental scaffold for this function. The degree of biological autonomy should therefore correlate with how completely the system internalizes information, transformation, and boundary maintenance. This can be tested by systematically withdrawing host-derived metabolites, replication machinery, membrane support, or environmental control and quantifying persistence and evolution. Table 6 summarizes the principal predictions together with the findings that would count against them.

## 10. Objections, Counterexamples, and Boundary Conditions

### 10.1. The Pattern-Matching Objection

The strongest objection is that any complex system can be divided into three parts if the investigator is sufficiently creative. The TPB accepts the force of this criticism and responds with prospective criteria, null models, and perturbational tests. A triad identified after the outcome is known is exploratory. Confirmatory evidence requires that the system boundary, domains, causal predictions, and alternative decompositions be registered before analysis. Examples selected because they already contain the number three cannot establish universality.

### 10.2. Dyads Can Regulate

Two-component bacterial systems [129], toggle switches [130], receptor–ligand pairs, and reciprocal feedback loops can regulate real biological processes. The slogan that a dyad can switch while only a triad can self-regulate is therefore too absolute, and it is abandoned. The strongest form of this objection is not a molecular module but a theory: Wicken derived from thermodynamics a two-function account of biological organization, exploitation and homeostasis, in which the maintenance of the self against environmental fluctuation is a single function rather than two [13]. The TPB’s defensible claim is accordingly not that dyads cannot regulate but that autonomous biological closure requires three broad functions because informational integrity and boundary integrity fail separately and recoverably, which a single homeostatic function cannot represent; Section 2.2 states the discriminating experiment and concedes that a negative result would return the framework to Wicken’s dyad. A bacterial two-component sensor and response regulator, meanwhile, operates within a cell that supplies genetic information, metabolism, membrane organization, and environmental context. The local dyad is genuine; it is not an autonomous biological system.

### 10.3. Larger Systems Are Not Counterexamples

Many systems require more than three components, modules, or control variables. The TPB does not claim otherwise. A tetradic or higher-order system challenges the TPB only if a fourth function is both irreducible and cannot be represented as an implementation, subdivision, or coupling of Information, Transformation, and Interface. The burden is symmetrical: proponents must not absorb every inconvenient function by definition, while critics must distinguish physical multiplicity from functional irreducibility. To make the proponent’s burden concrete, absorption is illegitimate whenever the candidate function can be selectively perturbed without perturbing any of the three domains, and whenever its removal degrades the emergent property more than the removal of any single domain. Either finding would establish a fourth domain, and Section 2.2 identifies the separation of energy transduction from material synthesis as the most likely place to find one. A second candidate deserves a direct answer. Replication is not obviously reducible to information storage, energy transformation or boundary maintenance, and Gánti listed reproduction among his potential rather than absolute criteria. The claim that every autonomous biological system replicates is, however, too strong as stated. Many individual organisms are sterile, post-reproductive or terminally differentiated, and reproduction in such cases is a property of the lineage or population rather than of the individual. Evolutionary theory itself treats this distinction as a matter of degree rather than of kind: what counts as the production of a new individual, and how strongly a collection of such individuals behaves as a Darwinian population, vary continuously across the living world [131]. The defensible claim is narrower—that a lineage capable of indefinite persistence and cumulative evolution requires a reproducible organization—and the TPB’s position is that replication is a capacity that closure makes possible rather than a fourth domain of the living state: a system must already possess informational, transformational and boundary functions in coordinated operation before it can produce a second such system. This is consistent with Gánti’s division between absolute criteria, which characterize being alive at a moment, and potential criteria, which characterize a living world persisting over time, and it converges with the metabolism-first side of the origin-of-life debate, on which the replication-first alternative makes selection more fundamental than organization and thereby leaves unexplained how bare replicators come to possess the organization that coded translation and self-maintenance require [13,20]. The position is defensible but not free: it predicts that no system will be found which replicates faithfully while lacking one of the three domains, and the discovery of such a system would require revision to a four-domain architecture. Obligate intracellular parasites and synthetic replicators are the natural places to look.

### 10.4. Specialized and Reduced Biological Systems

Mature mammalian erythrocytes lack nuclei and mitochondria yet remain viable for a limited period. Platelets lack nuclei. Endosymbionts and parasites can lose metabolic and biosynthetic functions, in extreme cases retaining genomes smaller than those of some organelles [132]. These cases are important boundary tests. The TPB interprets them as specialized, developmentally produced, and system-dependent units that borrow information or material support from a larger organism. This interpretation may not rest on the TPB’s own vocabulary. The classification is made by the withdrawal measure in Section 2.5, registered before the system is examined, and a reduced system that scored as autonomous on this measure would stand as a counterexample and could not be reclassified as scaffolded after the fact. The relevant question is whether the isolated entity is autonomous over the timescale and function being claimed. If a nucleus-free cell can persist indefinitely, self-repair comprehensively, and reproduce without external informational support, it would be a strong counterexample.

### 10.5. Universality Across the Tree of Life

The current evidence is strongest for cellular autonomy and vertebrate physiological systems. Plants, fungi, microbial communities, colonies, and ecosystems may instantiate the domains differently. The TPB should not assume identical anatomy or scale mappings. Its universality must be earned through comparative biology. The theory will become more credible by predicting in advance how distributed regulation in plants, communal metabolism in microbes, or supraorganismal boundaries in colonies should be analyzed—and by accepting revision if the three-domain decomposition fails.

### 10.6. Broader Applications Beyond Biology

Triadic reasoning may illuminate non-biological systems, including relations among a generative source, an organized substrate, and a regulating or mediating factor. Such analogies may be intellectually useful, but they do not constitute evidence for the TPB. The principle should stand or fall on biological data. A separate general theory of triadic organization could later examine physical, social, or astronomical systems using domain-specific criteria. Keeping these projects distinct protects the scientific identity of the TPB.

## 11. Conclusions: From Recurrence to Research Program

The Triadic Principle of Biology proposes, as a hypothesis, that living organization may repeatedly converge on closure among three functionally distinguishable domains: Information, Energy/Material Transformation, and Interface/Boundary. The proposal is broader than the observation that groups of three occur in textbooks and narrower than the claim that the universe is governed by a biological law. It concerns autonomous or self-regulating biological systems, their emergent properties, and the ways in which these properties fail and recover.

The evidence is heterogeneous and must be weighted rather than summed. The triplet codon is an exact conditional minimum about code word length, not functional domains, and should carry no weight. Translation provides a template–adaptor–catalytic core embedded in a larger apparatus; minimal-cell theory and Gánti’s chemoton provide a historical precedent proposed on independent grounds; Wicken’s argument that organized autocatalytic systems precede heritable templates converges on Section 10.3, though his wider program was judged to yield no testable hypothesis [26], which is the standard against which these predictions should be measured. Cellular physiology is consistent with reciprocal dependence among the three functions without reducing them to three organelles. The graph-theoretic, control-theoretic and dynamical arguments do not support the framework at all and are retained only to record why. Numerically attractive but mechanistically weak mappings should not carry the theory. Nothing assembled here shows that three domains are biologically privileged; this is the hypothesis, not a finding.

The disease extension may be the most consequential, and it is the least tested. On the proposed model, a disturbance can begin in one domain, propagate through couplings, exhaust compensation, and produce triadic disruption. This framework generates a pathology vocabulary—deficiency, hyperactivation, hijack, bypass, coupling failure, compensatory exhaustion, and collapse—and three corollaries, each a prediction awaiting test and none a result. Diagnosis is predicted to be able to identify the first-failing domain and loss of coordination prospectively. Biomarker panels should be tested across domains rather than assumed superior because they contain three measurements. Therapy is predicted to be more durable when it restores the functions and relationships necessary for closure, whether achieved by one intervention or several. None of these disease formulations is validated by the existing literature.

The status of this paper should be stated without ambiguity. What is offered is a candidate architecture, an explicit rule for assigning processes to domains and for fixing the system boundary, a definition of autonomy that does not presuppose the framework, a vocabulary for failure, an operationalized index with stated scoring functions and a worked example in the Supplementary Materials, and a specification of the observations that would require the principle to be abandoned. The examples surveyed in Section 3, Section 4, Section 5 and Section 6 are motivating cases; several fail the criteria in Table 1 and are recorded as failures in Table 4, since a framework that accommodated every example would not be worth testing. TPB will not be established by the number of examples assembled in its favor. It will be established, revised, or rejected by prospective model comparison against dyadic, tetradic and rival triadic alternatives, by perturbation, by comparative biology, by synthetic-cell experiments, and by independent disease validation conducted by groups other than the author’s. The decisive question for any new biological system is therefore not “Where is the triad?”; it is whether a predefined triadic functional architecture explains necessity, coupling, emergence, and failure better than the alternatives. If the answer repeatedly proves to be yes, the triadic hypothesis will have earned consideration as an organizational principle of life. If it proves to be no, the framework should be revised or abandoned as Section 3.4 specifies.

## Figures and Tables

**Table 1:** Prospective criteria distinguishing a genuine TPB triad from an arbitrary grouping.

Criterion	Operational Question	Exclusion Rule
Defined system boundary	What entity, scale, and time window constitute the system?	Reject mappings whose boundary changes to preserve a triad.
Defined emergent property	Which system-level property is claimed to arise from the triad?	Reject examples that list components without an emergent outcome.
Functional differentiation	Do the three domains perform distinguishable causal roles?	Reject synonyms or subdivisions of one process.
Non-substitutability	Can another domain fully replace the perturbed domain under the specified conditions?	If two domains are functionally interchangeable, the proposed triad is not irreducible.
Reciprocal coupling	Does each domain causally influence, directly or indirectly, the other two?	Simple serial chains without feedback are not closed triads.
Organizational closure	Do the domains jointly maintain the conditions of their own continued operation?	Co-occurrence without mutual maintenance is insufficient.
Perturbational necessity	Does selective disruption impair the defined emergent property in a domain-specific manner?	Association alone does not establish a TPB node.
Prospective and comparative testing	Does the triadic model outperform dyadic, tetradic, and unconstrained alternatives?	Post hoc relabeling after seeing the outcome is not confirmatory evidence.

**Table 3:** Behavior of the generalized mean *M_p_* over the three domain terms. For nonnegative domain scores, complete loss of one domain leaves *M_p_* positive for *p* > 0 if another domain remains positive, whereas the continuous extension for *p* ≤ 0 gives *M_p_* = 0 whenever any domain score is zero. Partial trade-off persists at every finite *p*. TPB’s claim is *p* ≤ 0, and the geometric mean (the *p* → 0 limit) used in this article is the least restrictive member of this family. The last two rows show why the minimum function, although it encodes non-substitutability most strictly, is unsuitable: it cannot distinguish a system with one severely impaired domain from one that is uniformly impaired.

Scenario	I, E, B	*p* = 1 Arithmetic	p → 0 Geometric	*p* = −1 Harmonic	p → −∞ Minimum
Balanced mild deficit	0.90, 0.90, 0.85	0.883	0.883	0.883	0.850
One domain halved	0.90, 0.90, 0.50	0.767	0.740	0.711	0.500
One domain severely impaired	0.95, 0.95, 0.20	0.700	0.565	0.422	0.200
One domain absent	1.00, 1.00, 0.00	0.667	0.000	0.000	0.000
All domains uniformly impaired	0.50, 0.50, 0.50	0.500	0.500	0.500	0.500

**Table 4:** Evidence matrix for representative candidate triads across scales. For each system the table states the emergent property claimed, the proposed domain assignment, the strongest competing decomposition, the evidence bearing on non-substitutability and reciprocal coupling, the current status of the mapping against the criteria in Table 1, and the observation that would falsify it. The purpose of the added columns is to prevent the table from being read as a list of confirmations: the entries differ substantially in evidential status, and two of them fail the criteria as currently formulated. These assignments are hypotheses, not license to force every system into a predetermined anatomical list.

Scale or System	Emergent Property Claimed	Domain Assignment—Information/Energy–Material/Interface–Boundary	Strongest Competing Decomposition	Evidence for Non-Substitutability and Coupling	Status Against Table 1, and the Observation that Would Falsify the Mapping
Minimal cell/chemoton	Autonomous persistence and self-reproduction of a chemical unit	Template replication; autocatalytic metabolism; membrane production and compartmentation	Gánti’s own five absolute criteria, which are not tripartite	Both stipulated by the model rather than measured: each subsystem’s product is another’s substrate [4,22].	Strongest candidate, but no chemoton has been synthesized. Falsified by a stable infra-biological system combining only two subsystems that matches chemoton persistence and evolvability.
Translation	Faithful, processive conversion of sequence into polypeptide	mRNA template; peptidyl transferase catalysis and factor-driven translocation; codon–anticodon decoding environment	Template–adaptor–catalyst, a different tripartition of the same system	No characterized system translates without a template, an adaptor and a catalytic center; decoding and catalysis constrain each other. Not tested at matched magnitude.	Passes differentiation and non-substitutability; fails one-to-one mapping, since rRNA carries both catalytic and interface roles. Retained as an illustration of a functional core, not as support. Falsified by a translation system in which one molecular species is dispensable without loss of fidelity.
Cell	Homeostasis, fate decision and recovery from perturbation	Genome and heritable regulatory state; metabolic networks and bioenergetics; cell envelope, transporters and receptors	Supply–demand–control; sensing–regulation–action	Inferred from the lethality of losing any one, not from matched perturbation. Coupling demonstrated for the nuclear–mitochondrial and membrane–metabolic axes [8,51,52].	Passes differentiation and reciprocal coupling. The primary test in Section 9.2. Falsified if a rival tripartition predicts perturbation outcomes as well under matched parameterization.
Epigenetic regulation	Stability, responsiveness and locus specificity of gene expression	DNA methylation as mitotically propagated memory; chromatin remodeling as ATP-dependent architectural work; non-coding RNA as sequence-specific targeting	Writers–readers–erasers; or a decomposition including 3D genome organization and RNA modification as separate irreducible layers	Neither established. The mechanisms interact bidirectionally [55,56,57,58,59], but compensation has not been tested for a defined locus and property.	Fails as currently formulated. The three named mechanisms are neither exhaustive nor cleanly separable, and the competing decomposition is at least as plausible. Recorded as a failure rather than absorbed.
Germ layers	Expansion of internal organization and locomotion in animals	No stable assignment in any of the three domains	Triploblasty as a developmental pattern with no functional tripartition implied	Neither applies: no assignment survives Table 1.	Fails. Each layer contributes to all three functions and no non-forced mapping exists. Included only to demonstrate that numerical coincidence is rejected.
Immune defense	Competent, self-limiting defense with memory	Adaptive memory and response selection; innate effector and inflammatory execution; barriers and antigen sampling	Recognition–effector–regulation, which maps the same system onto different boundaries	Barrier loss is not compensated by intact innate or adaptive function, or conversely, but not at matched magnitude. Coupling established in both directions [60,61].	Provisional. Each branch contains all three functions internally, which makes the coarse-graining difficult to falsify. Requires the perturbational test in Section 9.2 before counting as support.
Complex disease	Self-sustaining pathological state resistant to single-target therapy	Regulatory programs and pathological memory; metabolic and redox capacity; barrier integrity, receptors, tissue–environment exchange	Established network medicine module decompositions; dynamical network biomarkers	Neither established. Metabolic, inflammatory and barrier coupling is documented; closure is not.	Untested. The framework’s principal clinical claim. Falsified if pre-specified triadic panels fail to outperform size-matched alternatives, or if durable recovery occurs despite persistent failure of a domain claimed indispensable.

**Table 5:** Proposed modes of pathological change within TPB, each with the measurable signature that would identify it and the prediction that distinguishes it from generic disease progression or undifferentiated network dysregulation. The modes are proposed descriptive categories, not established disease mechanisms; the third and fourth columns exist so that each can be tested rather than merely applied as a label.

Mode	Definition	Illustrative Interpretation	Measurable Signature	Prediction Distinguishing It from Generic Disease Progression
Domain deficiency	A necessary function falls below the viable range.	Energy failure during ischemia; impaired barrier integrity; loss of regulatory information.	One domain term falls while the other two and all coupling terms remain within reference.	Restoring the deficient domain alone should restore Θ and the emergent property, with no residual coupling abnormality.
Domain hyperactivation	A normal function exceeds its appropriate magnitude or duration.	Persistent inflammatory transcription or excessive oxidative execution.	One domain term falls through the upper rather than lower limb of the viability score, with the raw indicator elevated.	Suppression toward the reference value, not further stimulation, should restore Θ—a directional prediction that an additive severity score cannot make.
Triadic hijack	An external or internal agent uses intact host machinery for a pathological program.	Chronic microbial products driving receptor–transcription–effector loops.	All three domain terms remain near reference while couplings are strengthened beyond reference and their lags shorten.	Removing the external driver should normalize couplings rapidly; suppressing any single host domain should not, and may worsen the emergent property.
Domain bypass	A process propagates without using the normal control route.	Protein conformational templating bypassing sequence change in prion propagation.	The emergent property degrades while the domain term for the bypassed control route remains within reference.	Interventions targeting the nominal control route should have no effect on Θ, whereas interventions on the bypass route should—a dissociation not predicted by severity models.
Coupling failure	The domains remain individually active but their timing, gain, or directionality becomes incoherent.	Metabolic output no longer matches signaling demand; receptor activation becomes uncoupled from resolution.	Coupling terms fall while all three domain terms remain within reference; Θ falls before any single marker crosses a conventional threshold.	The central and sharpest prediction of the framework. Detectable only by longitudinal cross-domain measurement, and invisible to any single-marker or additive panel.
Compensatory exhaustion	Initially protective responses become insufficient or maladaptive under prolonged demand.	Chronic endocrine, immune, or metabolic compensation.	A domain term maintained near reference at the cost of a progressively rising coupling deviation, followed by abrupt joint decline.	Rising variance and lengthening recovery time should precede the decline, giving a testable early-warning signature comparable to critical-transition indicators.
Triple-node collapse	All three domains and their couplings lose the capacity to restore the defined emergent function.	Terminal systems failure in severe degenerative or multiorgan disease.	All domain and coupling terms below reference simultaneously; Θ approaching zero with demonstrated hysteresis.	Restoration of any single domain should fail to restore the emergent property, distinguishing collapse from severe but recoverable deficiency.

**Table 6:** Principal falsifiable predictions of TPB, with the design required to test each and the finding that would count against the framework.

Prediction	Experimental Design	Finding that Would Challenge TPB
Triadic motif enrichment	Annotate domains prospectively; compare observed closed triads with function- and degree-preserving null networks.	No enrichment, or equal/better enrichment of dyadic or tetradic motifs at the same control points.
Functional irreducibility	Perturb each domain and coupling; measure emergent function, recovery, and robustness. Comparators must include the dyadic exploitation–homeostasis scheme and the sensing–regulation–action and supply–demand–control tripartitions, each fixed in advance and matched for parameter count.	A two-domain system reproduces the full function and resilience of the proposed triad.
Multi-domain biomarkers	Compare pre-specified triadic panels with all one- and two-domain panels, random triplets, and unconstrained models. Size-matched alternatives must include panels drawn from the rival tripartitions in Section 2.2.	Triadic panels do not generalize or add information beyond matched alternatives.
Restoration of closure	Test upstream, single-domain, sequential, and coordinated interventions with dynamic mediation analysis.	Durable recovery occurs despite persistent failure of an allegedly indispensable domain or coupling.
Autonomy–dependency relationship	Withdraw external support from synthetic cells, parasites, symbionts, and viruses.	A persistent, evolvable autonomous system lacks one proposed functional domain.
Cross-scale nesting	Map TPB domains at multiple stratodynamic layers and test upward/downward coupling.	Domain assignments are unstable, non-predictive, or require post hoc relabeling at each scale.

## Data Availability

No new empirical datasets were generated or analyzed in this theoretical study. The equations and illustrative numerical examples are provided in the article and Supplementary Materials.

## Supplementary Materials

The following supporting information can be downloaded at https://www.mdpi.com/article/10.3390/biology15211966/s1: Document S1: Section S1, Antecedent frameworks and the historical record; Table S1, Comparison of TPB with four antecedent frameworks on eight dimensions fixed in advance; Section S2, Worked computation of the closure index in the periparturient dairy cow; Table S2, Worked computation of the closure index. References [2,3,4,5,6,9,13,22,23,24,25,26,27,28,42,43] are cited in the Supplementary Materials.

## Abbreviations and Glossaries

The following abbreviations and glossaries are used in this manuscript:

**Abbreviations**	
**Abbreviation**	**Definition**
AMPK	AMP-activated protein kinase
mTORC1	Mechanistic target of rapamycin complex 1
NLRP3	NOD-like receptor family pyrin domain-containing 3
TPB	Triadic Principle of Biology
**Glossary of Technical Terms**	
**Term**	**Definition**
**Organizational closure**	The condition in which the parts of a system jointly maintain the conditions each requires in order to keep operating. A statement about organization, not about exchange: living systems are thermodynamically open.
**Emergent property**	A property belonging to a whole system and to none of its parts individually, which disappears when the system is taken apart. Operationalized here by selective perturbation.
**Functional domain**	A category of biological work, which may be carried out by many physical components. Distinct from a protein domain or a domain of life.
**Non-substitutability**	The condition that no amount of activity in the remaining domains can replace a failed one.
**Reciprocal coupling**	Two domains each influencing the other, directly or indirectly, rather than one acting on the other in a single direction.
**Integrator**	A molecule or process acting across all three domains, such as AMPK, mTORC1 or intracellular Ca^2+^. An integrator is not a domain.
**Closure index (Θ)**	A proposed 0–1 summary of whether the three domains are individually intact and still effectively coupled (Box 2).
**Coupling failure**	A state in which domains remain individually within range but the timing, gain or direction of their mutual influence becomes incoherent.
**First-failing domain**	The domain or coupling in which a disease process begins, before the disturbance propagates to the others.
**Chemoton**	Gánti’s model of a minimal living unit: a metabolic cycle, a self-copying template and a membrane, coupled stoichiometrically.
**Autopoiesis**	The proposal that a living system continuously produces the network of processes that produces it.
**Infra-biological system**	A system combining only two of the three chemoton subsystems, studied as a possible evolutionary route to full closure.
**Network motif**	A small wiring pattern occurring far more often in a real network than in a randomized network of the same size and connectivity.
**Null model**	A randomized version of a system preserving every feature except the one being tested, used to establish what would be expected by chance.
**Degree-preserving randomization**	A null model in which every node retains its original number of connections.
**Requisite variety**	Ashby’s principle that a regulator must be able to occupy at least as many distinct states as the disturbances it must counter.
**Feed-forward loop**	A three-node circuit in which A regulates B and both regulate C, allowing the output to respond to sustained but not transient signals.
**Limit cycle**	A self-sustaining oscillation, such as a circadian rhythm.
**Bifurcation**	The point at which a small further change in conditions makes a system switch to qualitatively different behavior.
**Hysteresis**	Failure of a system to recover along the path by which it was disturbed, so that improvement lags behind removal of the cause.
**Size-matched comparison**	A rival model containing the same number of measurements, so that any advantage cannot be attributed to measuring more things.
**Mediation analysis**	A statistical test of whether an intervention achieves its effect through a proposed intermediate step.
**Stratodynamics**	A companion framework describing how biological responses are organized across layers from molecule to organism, each with its own timescale.
**Functional integrity score**	A 0–1 transformation of a raw indicator in which 1 denotes operation within the viable range and both deficiency and excess score lower. Required for the domain terms of Θ.
**Viable reference pattern**	The direction, gain, timing and proportionality of a coupling in a healthy system, against which observed coupling integrity is scored.
