# Supplementary material for: The Triadic Principle of Biology: A Testable Hypothesis About Functional Closure in Living Systems
Source: Biology (Basel). 2026 Nov 1;15(21):1966. doi: 10.3390/biology15211966 (PMC13761747; doi:10.3390/biology15211966)
Supplement: Supplementary file 1 [file biology-15-01966-s001.zip › biology-4537170-supplementary.pdf]

Supplementary Materials

The Triadic Principle of Biology: A Testable Hypothesis About Functional Closure in Living Systems

Burim N. Ametaj

Department of Agricultural, Food and Nutritional Science, Faculty of Agricultural, Life and Environmental Sciences, University of Alberta, Edmonton, AB T6G 2P5, Canada; bametaj@ualberta.ca

This document contains material moved from the main article at the request of the Academic Editor: the historical treatment of antecedent frameworks (Section S1, formerly part of Section 2.8) and the worked computation of the closure index (Section S2, formerly part of Section 3.3). The text is unchanged apart from cross-references. Section, table and figure numbers without the prefix S refer to the main article, and reference numbers refer to the main article's reference list; the references cited here are listed at the end of this document with their main-article numbers.

Section S1. Antecedent Frameworks and the Historical Record

Tibor Gánti's chemoton is the closest formal antecedent and should be described precisely. It models a minimal living unit as three stoichiometrically coupled autocatalytic subsystems: an open metabolic cycle, a self-replicating template polymer, and a membrane enclosing both [4,22]. The 1971 formulation contained the metabolic and template cycles, and the membrane was added as a formal third subsystem in 1974 [23]. The correspondence with Energy/Material Transformation, Information, and Interface/Boundary is genuine and is not disputed here. Autopoiesis likewise emphasizes a self-producing network enclosed by a boundary [5]; its closure is operational rather than stoichiometric, in that the network produces the components that produce the network, and the boundary is among the products rather than a separately maintained function. Rosen's relational biology emphasizes metabolic and repair entailments and closure to efficient causation [6], and is more abstract still: no boundary term appears in the formalism, and the result is demarcational, separating organisms from mechanisms rather than grading either. Closure-of-constraints theory locates closure at the level of constraints, each maintained by at least one other while the system remains open to matter and energy [2,3]. These three frameworks and the chemoton are set against TPB on fixed dimensions in Table S1.

One feature of Gánti's system deserves emphasis because it is often overlooked, including by those who invoke the chemoton as a precedent for triadic thinking. Gánti's definition of the living state does not rest on three of anything. His absolute life criteria number five — inherent unity, metabolism, inherent stability, an information-carrying subsystem, and program control — with a further set of potential criteria covering growth, reproduction, heritable change and mortality [4]. The three coupled subsystems are his chemical implementation of those criteria, not the criteria themselves. This distinction matters for the present argument in a way that favors neither party automatically: it shows that Gánti arrived at a tripartite architecture while working from a five-part definition, which is evidence that the tripartition is architectural rather than definitional, and it also shows that the number three was never Gánti's thesis.

A second antecedent was drawn to the author's attention during review. It should be recorded in two parts, because Wicken's conceptual work and his wider evolutionary program were received very differently and only the first is relied on here. The conceptual work is careful and is used directly in Section 2.1: Wicken argued that Shannon entropy does not generalize thermodynamic entropy, that a specified sequence is a completed structure with zero configurational entropy rather than an ensemble,

that what the Shannon formula measures is complexity of structural relationships, and that order, complexity and information are three distinct properties that the shared formalism has encouraged biologists to conflate [9,24]. He put the same point concretely: organisms are highly complex at the level of macromolecular constituents and highly ordered at the level of statistical ensembles, and the freezing of water is an ordering rather than an information-generating process [24]. That argument is the reason this article states that the Information domain is not restricted to Shannon entropy, and it is a stronger warrant for the claim than the assertion it replaces.

The wider program proposed that thermodynamics supplies the foundational principles for both the origin of life and its subsequent evolution [13,25]. The correspondence with TPB is not a third tripartition: Wicken's thermodynamics is foundational rather than one of three co-equal aspects, and his own functional decomposition is dyadic — exploitation and homeostasis, treated as a rival in Section 2.2 [13]. Nor does he lack a boundary concept; what he lacks is a separately maintained boundary function, the boundary being for him a consequence of homeostatic activity rather than a domain requiring upkeep [13], and that is the substance of the disagreement rather than a matter of counting. What is genuinely parallel is narrower and more useful: Wicken rejected the primal-replicator account on the ground that selection on rate and fidelity cannot explain how replicators acquire the organization coded translation requires, defined organisms as autocatalytic organizations rather than replicators, and treated genetic information as part of that network rather than as a program standing outside it [13,25]. That is the position of Section 10.3 — replication as a capacity closure makes possible rather than a fourth domain — reached from thermodynamics rather than from pathogenesis, and it is recorded as a convergence rather than as support.

The criticism that program attracted matters here more than its content. Reviewing the book, Ayala judged its account of organismal evolution opaque and objected that thermodynamic considerations "yield no specific testable hypothesis that might guide experiment and observation" [26]. The irony sharpens the lesson: Wicken had opened his 1980 paper by faulting neo-Darwinism for weakness against precisely the criteria of prediction and falsifiability [13]. That is the failure mode a broad organizational framework most readily invites, and it is the one this article is built to avoid — which is why Section 9 states five predictions with the findings that would count against each, why Section 3.3 gives the closure index a computational procedure rather than a restatement of its own definition, and why Section 3.4 sets out the conditions under which TPB should be abandoned rather than repaired.

What the chemoton is determines what it can be an antecedent for. It is a model of the minimum chemistry sufficient for something to count as alive: a contribution to the origin-of-life problem and to the theory of minimal cells. Its domain of application is a single, undifferentiated, self-reproducing chemical unit, and its three subsystems are abstract chemical cycles rather than cellular structures. It makes no claim about tissues, organs, physiological regulation, immune organization, development, or the failure of any of these, and it was not designed to. Griesemer has argued that the chemoton and Gánti's life criteria are best understood as a heuristic platform for constructing an exact theory of minimal life rather than as a description of extant cells [23], a reading that further clarifies the boundary of its intended domain. Subsequent work has analyzed infra-biological systems combining any two of the three subsystems as possible evolutionary routes to full chemoton organization [27,28]; these provide named candidate systems for the perturbational test of Section 9.2, in which a two-domain system that achieved the same emergent property and robustness as the proposed triad would count against TPB.

S1.1. Comparison with Four Antecedent Frameworks

A comparison of this kind is useful only if the dimensions are fixed before the entries are written, rather than chosen so as to flatter the newer framework. Table S1 therefore sets the four antecedents most often proposed as precedents against TPB on eight dimensions declared in advance: the unit of analysis, the meaning of closure, the role of boundaries, the treatment of information, whether metabolism is necessary, whether replication is required, whether the framework is descriptive or normative, and whether it yields quantitative predictions.

Two differences run through the table, and the second is not wholly in TPB's favor. The first concerns the boundary. Each antecedent treats it either as something the rest of the organization produces (autopoiesis, closure of constraints), as one subsystem among three whose role is containment (the chemoton), or as absent from the formalism altogether (relational biology). TPB treats it as a separately maintained function that can fail while the other two remain within range, which is what makes barrier failure a distinct category rather than a downstream sign of metabolic failure. The second is that TPB alone grades closure continuously instead of using it to sort systems into the living and the non-living. That is what permits failure modes and quantitative predictions to be attached to it, and it is also its principal liability, since a graded index has parameters and parameters can be tuned. Sections 3.3 and 3.4 exist for that reason, and the framework should be judged partly on whether those safeguards hold.

Table S1. Comparison of TPB with four antecedent frameworks on eight dimensions fixed in advance. The entries state what each framework claims, not what it could be extended to claim; where a framework is silent on a dimension this is recorded as silence rather than as disagreement.

**Dimension**	**Chemoton (Gánti) [4,22,23]**	**Autopoiesis [5]**	**Relational biology (Rosen) [6]**	**Closure of constraints [2,3]**	**TPB (this article)**
**Unit of analysis**	One minimal, undifferentiated self-reproducing chemical unit; abstract cycles, not cellular structures.	The individual living unity, typically the single cell.	The organism as a formal entailment structure, abstracted from material realization.	The organism as a set of constraints acting on the processes that produce them.	Any system at any scale, with its boundary and emergent function declared in advance.
**Meaning of closure**	Stoichiometric: each subsystem's products are the others' substrates.	Operational: the network produces the components that produce the network.	Closure to efficient causation: every efficient cause is produced within.	Constraints mutually maintain one another, while the system stays open to matter and energy.	Each domain maintains the conditions the other two require. Graded, not binary; quantified by Θ(t).
**Role of boundaries**	A co-equal subsystem: a membrane grown from a metabolic precursor (added 1974).	Produced by the network and necessary to it, but not separately maintained.	Absent from the formalism; there is no boundary term.	One constraint among others; not privileged.	A co-equal domain, separately maintained, able to fail alone. A function, not a membrane.
**Treatment of information**	A template polymer whose copy number sets division; no semantic content is claimed.	Explicitly rejected; the unity is described without information or representation.	Not a primitive; entailment relations take its place.	Rendered as the specificity of a constraint; no informational domain.	A co-equal domain: distinctions that alter behavior. Not Shannon entropy, not DNA alone (Section 2.1).
**Is metabolism necessary?**	Yes. An autocatalytic metabolic cycle is one of the three subsystems.	Yes. Production of the system's own components is constitutive.	Yes. Metabolism is one of the two mappings of an (M,R)-system.	Yes. Closure is realized over metabolic processes.	Yes, but broader than metabolism: mechanical work and regulated degradation are included.
**Is replication required?**	Yes. Template replication and division are internal to the model.	No. Reproduction follows from autopoiesis rather than conditioning it.	Not assumed; replication is derived within the formalism.	No. It forms no part of the definition.	No. A capacity closure makes possible, not a fourth domain (Section 10.3).
**Descriptive or normative?**	Normative for a stipulated minimal unit: criteria for what counts as alive.	Definitional. A binary criterion for life; systems are not graded.	Definitional. It demarcates organisms from mechanisms.	Descriptive. An account of how organisms are organized.	Both. Descriptive of architecture, normative about failure; grades closure and names failure modes (Table 7).
**Quantitative predictions?**	Quantitative in its own kinetics, but predicts nothing about extant organisms; none has been synthesized.	None. There is no measure of degree of autopoiesis.	None empirical; the formal results are themselves contested.	None. The framework is conceptual.	A closure index with a stated estimation procedure (Section 3.3) and five falsifiable predictions (Section 9), all untested.

Section S2. Worked Computation of the Closure Index in the Periparturient Dairy Cow

The registration below is illustrative and its numerical values are hypothetical, chosen to demonstrate the computation rather than reported from data; no Θ value in this article is derived from measurement. Indicators, viable intervals and reference values are fixed in advance as follows. Interface/Boundary is represented by ionized calcium, viable interval 1.10–1.30 mmol/L with reference 1.20, evaluated specifically as an index of membrane excitability and secretory competence and not as a general marker of calcium biology. Energy/Material Transformation is represented by β-hydroxybutyrate, one-sided with upper viable limit 1.0 mmol/L, and non-esterified fatty acids, one-sided with upper viable limit 0.60 mmol/L. Information is represented by haptoglobin, upper limit 0.30 g/L, and serum amyloid A, upper limit 30 mg/L, read as the regulatory output of the inflammatory control program. This is the weakest assignment in the registration and is declared weak in advance. Acute-phase proteins are hepatic and inflammatory outputs, not measures of informational integrity: they are several steps downstream of the regulatory decisions they are being used to index, they respond to stimuli that have nothing to do with the coupling under test, and their concentration reflects hepatic synthetic capacity as much as regulatory state [42,43]. They are used here because they are measurable in the field at the required frequency, which is a practical reason and not an evidentiary one. If the Information term is the domain term that fails to behave as predicted, the first interpretation to be excluded is that the indicators were wrong rather than that the framework was. Essential couplings are set to $\mathcal{E}$ = all six directed couplings, m = 6, by the default rule of Section 3.3. κ = 6, $\alpha = \beta = 1$. Table S2 reports the resulting computation.

Table S2. Worked computation of the closure index in the periparturient dairy cow. Values are hypothetical and illustrate the computation; none is derived from measurement. The decisive comparison is between the two rows at day −3. Every individual indicator in the cow that later develops disease lies within its viable interval, and an additive score over the same three domain terms separates the two animals by 0.03, which no clinical protocol would act upon. The closure index separates them by 0.29, because the coupling term has already fallen. This dissociation is the sharpest testable claim the framework generates, and Section 9.3 states the study that would confirm or refute it. For the four one-sided indicators the reference value is $x^* = 0$, so that $d(x) = x / x_{hi}$; for ionized calcium, which is two-sided, x^* is the midpoint of the viable interval. The coupling terms in the table are the aggregate Θ inputs; the underlying directional estimates, their selected lags and the reference-cohort values (ϱ^*, ℓ^*, σ_ϱ, σ_ℓ) would be reported in full in any empirical application, and are omitted here only because the values are hypothetical.

Quantity (registered viable interval)	Day −3<br>remains healthy	Day −3<br>later diseased	Day +3<br>early clinical disease
**Ionized calcium, mmol/L (1.10–1.30, ref. 1.20)**	1.19	1.18	1.08
**β-Hydroxybutyrate, mmol/L (≤ 1.0)**	0.50	0.60	1.20
**Non-esterified fatty acids, mmol/L (≤ 0.60)**	0.40	0.45	0.72
**Haptoglobin, g/L (≤ 0.30)**	0.12	0.16	0.36
**Serum amyloid A, mg/L (≤ 30)**	14	18	38
**B — Interface/Boundary integrity**	1.00	0.99	0.23
**E — Energy/Material integrity**	0.92	0.87	0.23
**I — Information integrity**	0.97	0.93	0.20
**Mean coupling integrity C (m = 6 directed)**	0.91	0.62	0.40
**Closure index Θ**	0.87	0.58	0.09
**Additive score over B, E, I (comparator)**	0.96	0.93	0.22

References (numbered as in the main article)

2. Mossio, M.; Montévil, M.; Longo, G. Theoretical principles for biology: Organization. Prog. Biophys. Mol. Biol. 2016, 122, 24–35. https://doi.org/10.1016/j.pbiomolbio.2016.07.005.

3. Montévil, M.; Mossio, M. Biological organisation as closure of constraints. J. Theor. Biol. 2015, 372, 179–191. https://doi.org/10.1016/j.jtbi.2015.02.029.

4. Gánti, T. The Principles of Life; Oxford University Press: Oxford, UK, 2003.

5. Luisi, P.L. Autopoiesis: A review and a reappraisal. Naturwissenschaften 2003, 90, 49–59. https://doi.org/10.1007/s00114-002-0389-9.

6. Rosen, R. Life Itself: A Comprehensive Inquiry into the Nature, Origin, and Fabrication of Life; Columbia University Press: New York, NY, USA, 1991.

9. Wicken, J.S. Entropy and information: Suggestions for common language. Philos. Sci. 1987, 54, 176–193. https://doi.org/10.1086/289369.

13. Wicken, J.S. A thermodynamic theory of evolution. J. Theor. Biol. 1980, 87, 9–23. https://doi.org/10.1016/0022-5193(80)90216-7.

22. Szathmáry, E. Founder of systems chemistry and foundational theoretical biologist: Tibor Gánti (1933–2009). J. Theor. Biol. 2015, 381, 2–5. https://doi.org/10.1016/j.jtbi.2015.04.037.

23. Griesemer, J. The enduring value of Gánti's chemoton model and life criteria: Heuristic pursuit of exact theoretical biology. J. Theor. Biol. 2015, 381, 23–28. https://doi.org/10.1016/j.jtbi.2015.05.016.

24. Wicken, J.S. Entropy, information, and nonequilibrium evolution. Syst. Zool. 1983, 32, 438–443. https://doi.org/10.2307/2413170.

25. Wicken, J.S. Evolution, Thermodynamics, and Information: Extending the Darwinian Program; Oxford University Press: New York, NY, USA, 1987.

26. Ayala, F.J. Review of Evolution, Thermodynamics, and Information: Extending the Darwinian Program, by J.S. Wicken. J. Soc. Biol. Struct. 1990, 13, 283–284. https://doi.org/10.1016/0140-1750(90)90014-W.

27. Griesemer, J.; Szathmáry, E. Gánti's chemoton model and life criteria. In Protocells: Bridging Nonliving and Living Matter; Rasmussen, S., Bedau, M.A., Chen, L., Deamer, D., Krakauer, D.C., Packard, N.H., Stadler, P.F., Eds.; MIT Press: Cambridge, MA, USA, 2009; pp. 481–512. https://doi.org/10.7551/mitpress/9780262182683.003.0022.

28. Szathmáry, E.; Santos, M.; Fernando, C. Evolutionary potential and requirements for minimal protocells. Top. Curr. Chem. 2005, 259, 167–211. https://doi.org/10.1007/tcc001.

42. Cray, C.; Zaias, J.; Altman, N.H. Acute phase response in animals: A review. Comp. Med. 2009, 59, 517–526.

43. Uhlar, C.M.; Whitehead, A.S. Serum amyloid A, the major vertebrate acute-phase reactant. Eur. J. Biochem. 1999, 265, 501–523. https://doi.org/10.1046/j.1432-1327.1999.00657.x.
